# Hemodynamic Analysis in an Idealized Artery Tree: Differences in Wall Shear Stress between Newtonian and Non-Newtonian Blood Models

**DOI:** 10.1371/journal.pone.0124575

**Published:** 2015-04-21

**Authors:** Jared C. Weddell, JaeHyuk Kwack, P. I. Imoukhuede, Arif Masud

**Affiliations:** 1 Department of Bioengineering, University of Illinois at Urbana-Champaign, Urbana, Illinois, 61801, United States of America; 2 Department of Civil Engineering, University of Illinois at Urbana-Champaign, Urbana, Illinois, 61801, United States of America; Colorado State University, UNITED STATES

## Abstract

Development of many conditions and disorders, such as atherosclerosis and stroke, are dependent upon hemodynamic forces. To accurately predict and prevent these conditions and disorders hemodynamic forces must be properly mapped. Here we compare a shear-rate dependent fluid (SDF) constitutive model, based on the works by Yasuda et al in 1981, against a Newtonian model of blood. We verify our stabilized finite element numerical method with the benchmark lid-driven cavity flow problem. Numerical simulations show that the Newtonian model gives similar velocity profiles in the 2-dimensional cavity given different height and width dimensions, given the same Reynolds number. Conversely, the SDF model gave dissimilar velocity profiles, differing from the Newtonian velocity profiles by up to 25% in velocity magnitudes. This difference can affect estimation in platelet distribution within blood vessels or magnetic nanoparticle delivery. Wall shear stress (WSS) is an important quantity involved in vascular remodeling through integrin and adhesion molecule mechanotransduction. The SDF model gave a 7.3-fold greater WSS than the Newtonian model at the top of the 3-dimensional cavity. The SDF model gave a 37.7-fold greater WSS than the Newtonian model at artery walls located immediately after bifurcations in the idealized femoral artery tree. The pressure drop across arteries reveals arterial sections highly resistive to flow which correlates with stenosis formation. Numerical simulations give the pressure drop across the idealized femoral artery tree with the SDF model which is approximately 2.3-fold higher than with the Newtonian model. In atherosclerotic lesion models, the SDF model gives over 1 Pa higher WSS than the Newtonian model, a difference correlated with over twice as many adherent monocytes to endothelial cells from the Newtonian model compared to the SDF model.

## Introduction

Coronary artery disease causes 1 of every 6 deaths in America and can result from inadequate blood supply to cardiac muscles [1]. Similarly, occlusive vascular diseases and disorders such as arteriosclerosis, dementia, stroke, and coronary artery disease can develop as a result of abnormal blood flow [2, 3]. It remains challenging to identify pre-symptomatic patients who are at high risk of developing vascular occlusions [4]. Therefore, to better develop preventive techniques against hemodynamic dependent diseases, novel risk estimation techniques are necessary [5]. Hemodynamic modeling is one such preventive technique that can be utilized to predict disease risk.

Hemodynamic simulations have been performed using patient-specific geometries reconstructed from imaging data [6, 7]. Patient-specific geometries capture anatomical complexity observed in physiology, offering promising approaches to advance personalized medicine in clinical assessment and diagnosis. Patient-specific studies have analyzed hemodynamic forces in aneurysms [8, 9], ventricular assist devices [10], and in the heart and aorta [11, 12]. Despite patient-specific studies such as these, the ability of the biofluid mechanics community to apply new methods to study, recreate, and validate patient-specific geometries has been severely limited. This problem arises because patient-specific geometries are not easily shared due to the containment of patient information. To circumvent this problem, we present a biologically relevant idealized femoral artery tree geometry for numerical method verification. This test geometry will allow researchers to ensure that physiologically relevant numerical simulations are obtained before progressing into patient-specific geometries.

Hemodynamic simulations have advanced the understanding of atherosclerotic lesion development and rupture and developed techniques to prevent these processes. Such studies have revealed that atherosclerotic lesion formation via low density lipoprotein accumulation preferentially occurs in arterial regions exhibiting low wall shear stress (WSS) [13–15]. Others have correlated low density lipoprotein accumulation with decreased WSS within arteries exhibiting increases in diameter [16–18]. Numerical simulations have revealed an increased atherosclerotic lesion rupture risk for lesions experiencing high WSS magnitudes in both patient-specific [19–21] and simplified geometries [22, 23]. Computer models have provided insight into preventing atherosclerotic lesion development by showing that the deposition of systemically injected nanoparticles into atherosclerotic lesions is highly dependent on the spatio-temporal WSS across these lesions [24–26]. While these studies have resulted in better insight into atherosclerotic lesion development and rupture, the dependency of WSS magnitude distributions across atherosclerotic lesions to the shear-rate dependent viscosity of blood is not well defined. We seek to uncover this relationship of WSS and blood viscosity using advanced computer models under both healthy and atherosclerotic conditions.

**Fig 1:**
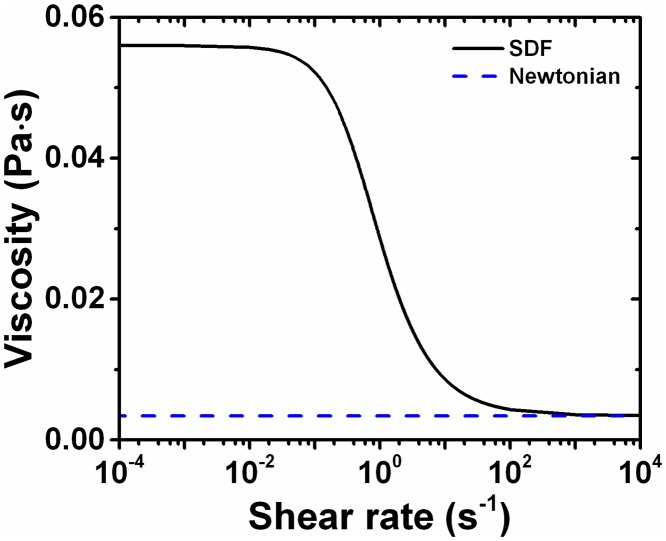
Viscosity of Newtonian and Carreau-Yasuda Constitutive Blood Models. The Carreau-Yasuda model of blood shows the viscosity changing as a function of the shear-rate. The Newtonian model has constant viscosity at all shear-rates.

Blood exhibits a range of viscosities dependent on the multiple cell types such as erythrocytes, leukocytes, and thrombocytes it contains. Blood viscoelastic characteristics are manifested via time-dependent strains in response to stress, primarily due to viscoelastic membrane deformation of erythrocytes [27–29]. Viscous stress of blood is also shear-rate dependent and undergoes shear-thinning, a decrease in viscosity with increasing shear-rate, primarily caused by transformation in microscopic rouleaux (stacks of erythrocytes) structures [30–32]. Various constitutive models of blood have been developed to capture this shear-rate dependency [33, 34]. Some commonly used non-Newtonian models for blood are the Power-Law, Casson, Herschel-Bulkley, and Carreau-Yasuda models [35, 36]. The Power-Law model displays a linear response between viscosity and shear-rate on the log-log scale that is not seen in experimental blood viscosity measurements [35]. While the Casson and Herschel-Bulkley models provide good fits to experimental viscosity measurements at intermediate to high shear-rates, they fail to accurately capture blood viscosity at lower shear-rates [36]. Others and we have shown previously that the model choice does lead to differences in hemodynamic results including velocity and wall shear stress profiles [35, 37, 38]. Due to the dependency of flow predictions to the model employed, we chose to use the Carreau-Yasuda model here [39] as it more accurately fits empirically measured blood viscosity than these other non-Newtonian models [40, 41] (Fig 1). Viscosity as a function of shear-rate given by the Carreau-Yasuda model is as follows:

$$\eta (\gamma )=\mu_{\infty }+(\mu_{0}-\mu_{\infty }){\left(1+{\left(\lambda \gamma \right)}^{a}\right)}^{(n-1)/a}$$ (1)

where *η* is the viscosity, *γ* is the shear rate, *μ*_*∞*_ is the viscosity at infinite shear-rate, *μ*_0_ is the viscosity at zero shear-rate, and *λ*, *α*, and *n* are material coefficients (*λ* = 1.902 s, *α* = 1.25, *n* = 0.22). For blood, *μ*_*∞*_ = 0.00345 Pa s, *μ*_0_ = 0.056 Pa s, and blood density is 1060 kg/m^3^. Henceforth, the Carreau-Yasuda model is referred to as the shear-rate dependent fluid (SDF) model.

Previous numerical simulations published in the literature have compared WSS between Newtonian and non-Newtonian models in coronary and femoral bypasses [42], curved artery models [22], 2-dimensional atherosclerosis [23], and patient-specific right coronary arteries [43]. While these studies have presented differences between Newtonian and non-Newtonian WSS, it remains unclear whether these differences are significant in a biological context, such as in determining atherosclerotic lesion growth rates. We attribute this deficiency to the lack of simple test geometries that would allow in-depth study of fundamental differences between Newtonian and non-Newtonian models to be observed in a biologically relevant context. The idealized femoral artery tree presented here is aimed to serve a a benchmark problem to reveal these fundamental differences.

This paper also explores whether the Newtonian assumption of blood can accurately capture the physics of blood flow or if non-Newtonian models are necessary. To address this question, we quantitatively compare blood velocity, pressure, and WSS between the Newtonian and SDF models first in an idealized context and then in a biologically relevant context. Specifically, we compare Newtonian and SDF models using the lid-driven cavity flow problem, described by Ghia *et al* [44], as this is a benchmark problem with well-established solutions published in the literature (see Supporting Information). We verify our stabilized finite element method employing the lid-driven cavity problem, as well as in a biologically relevant context using the idealized single artery bifurcation presented by Chung [45]. We present an idealized femoral artery tree as a biologically relevant test geometry and use it to compare the Newtonian and SDF models under both healthy and atherosclerotic conditions.

## Materials and Methods

### A stabilized finite element method for shear-rate dependent fluids

Stabilized finite element methods for non-Newtonian biological fluids were presented in Masud and Kwack [33, 35, 46–47]. These works are extensions of stabilized formulations for the Navier-Stokes equations by Masud and coworkers in [48–50]. Viscoelastic characteristics of blood at low shear-rates are markedly different from those at higher shear-rates and have the propensity to cause blood coagulation. Consequently, there has been tremendous interest in developing high fidelity numerical methods with enhanced stability properties [45, 47]. Specifically, the viscosity of blood varies substantially in the intermediate range of shear-rates that are typically encountered in human vasculature (Fig 1). To address this issue, a stabilized formulation for shear-rate dependent blood flow was developed in [33, 35] and extended to higher-order 3-dimensional elements in [46]. These studies [33, 35, 46–50] describe the complete formulations of the numerical method employed in this study.

To keep the discussion self-contained, we briefly describe the underlying method and the procedure. First, the desired geometry is discretized into a finite element mesh, and then stabilized finite element method for shear-rate dependent non-Newtonian fluids is employed for numerical simulations. The non-Newtonian viscous stress ***σ***_*v*_ is defined as follows:

$$\sigma_{v}=2\eta \left(\gamma \right)\varepsilon \left(v\right)$$ (2)

where *γ* is the shear-rate, *η*(*γ*) is the shear-rate dependent viscosity, and ***ε***(***ν***) is the rate-of-deformation tensor. The fluid flow is governed by the incompressible momentum balance equations where the shear-rate dependent viscous stress is fully embedded:

$$\rho v_{,t}+\rho v\cdot \nabla v-\nabla \cdot \sigma_{v}\left(v\right)+\nabla p=f$$ (3)

​​​​∇⋅v=0 (4)

Eqs (3) and (4) are the momentum-balance and incompressibility equations respectively, where *ρ* is the density, *ν* is the velocity field, *ν*_*t*_ is the time derivative of the velocity, *p* is the pressure field, and *f* is the body force.

Under suitable assumptions [33,35,46] the velocity field described by the incompressible momentum balance equations is decomposed into coarse- and fine-scales via the Variational Multiscale method [33, 46], leading to two sets of sub-problems that govern coarse- or resolvable scales and fine- or subgrid scales, respectively. The fine-scales are assumed to be represented by piecewise polynomials of sufficiently high order, continuous in space but discontinuous in time. It has been shown in our earlier works that the fine-scale problem is driven by the residual of the Euler-Lagrange equations for the coarse-scale. The fine-scale solutions thus approximate part of the physics that is beyond the resolution capacity of coarse-scale equations. These fine-scale solutions are then variationally embedded in the coarse-scale formulations, thereby resulting in the stabilized method. Solution of the stabilized form of the problem yields the coarse-scale solution on the given discretization, yet it accounts for the fine-scale features in the solution via the variationally embedded fine-scale model represented by the stabilization terms. Thus, the resulting solution possesses enhanced accuracy and stability properties and is variationally consistent.

This numerical method was implemented for linear and quadratic 3-dimensional elements and verified for stability and accuracy properties via numerical convergence-rate tests and steady-state/transient benchmark problems [33, 46]. For completeness of discussion, the resulting nonlinear stabilized finite element formulation is presented:

$$\begin{array}{l} \rho \left(w,v_{,t}\right)+\rho \left(w,v\cdot \nabla v\right)+\left(\nabla w,2\eta \varepsilon \left(v\right)\right)-\left(\nabla \cdot w,p\right)\\ \;\;\;\;\;\;\;\;\;\;\;\;\;+\left(q,\nabla \cdot v\right)-\left(\chi ,\tau \;r\right)=\left(w,f\right)+{\left(w,h\right)}_{\Gamma_{h}} \end{array}$$ (5)

where ***w*** the velocity field weighting functions, *η* is the element viscosity based on the mean value of the coarse-scale shear-rate, *q* is the pressure field weighting functions, ***h*** is the vector of the prescribed boundary tractions, and Γ is the boundary. The first five terms on the left-hand side of Eq (5) represent the standard Galerkin form of the incompressible momentum balance equations in (2) and (3). The last term on the left-hand side represents the stabilization terms (***χ*, *τr***) that are derived via a local solution of the fine-scale problem that is then embedded in the coarse-scale formulation. Specifically, the various stabilization terms in (5) are defined as follows:

χ=ρ(−∇v⋅w+(∇⋅v)w+v⋅∇w)+η(∇(∇⋅w)+Δw)+∇η⋅((∇⋅w)1+∇w)+∇q (6)

$$\tau =b^{e}{\left[\begin{array}{l} \rho \int {\left(b^{e}\right)}^{2}{\left(\nabla v\right)}^{T}\;d\Omega +\rho \int b^{e}v\cdot \nabla b^{e}\;d\Omega \;I\\ \;\;\;+\int \eta \left(\nabla b^{e}⊗\nabla b^{e}\right)\;d\Omega +\int \eta {\left|\nabla b^{e}\right|}^{2}\;d\Omega \;I \end{array}\right]}^{-1}\int_{\Omega^{e}}b^{e}d\Omega$$ (7)

$$r=\left(-\rho v_{,t}-\rho v\cdot \nabla v+2\eta \nabla \cdot \varepsilon \left(v\right)+2\nabla \eta \cdot \varepsilon \left(v\right)-\nabla p+f\right)$$ (8)

where *b* ^*e*^ are bubble functions, Ω is the open bounded domain, which is discretized into non-overlapping element domains Ω^e^, and ***I*** is the identity matrix. ***χ*** is the weighting function for the stabilization term given in (5). **τ** is the stabilization tensor comprised of the operators of the momentum balance equations acting on the fine-scale fields. It is numerically approximated via bubble functions and its gradients. ***r*** is the residual of the Euler-Lagrange equations for the coarse-scales over the sum of element interiors. Consequently, the resulting method is residual based which is an important consideration in ensuring the consistency of the method. Furthermore, the additional stabilization terms play a critical role in obtaining stable solutions for the highly pulsatile flows in biofluid dynamics.

The method employed here includes the fundamental features of classical stabilized methods (e.g., Streamline Upwind/Petrov-Galerkin (SUPG) method [51], or Galerkin/Least-Square (GLS) method [52]). In [48] it was shown that both SUPG and GLS are sub-classes of this method. In our earlier works, the enhanced stability of the method permitted us to use a first-order approximation for the nonlinear viscosity field without losing accuracy [35]. In [33] the method was extended to 3D employed hexahedral and tetrahedral elements with full description of nonlinear viscosity field. These methods [33, 35] come equipped with consistent mass/tangent tensors for nonlinear solution processes; therefore, they provide faster numerical convergence for large-scale transient simulations such as those presented here.

### Idealized femoral artery tree

The idealized femoral artery tree presented here contains rigid walls and straight cylindrical arteries and symmetric bifurcations. The mean flow rates [53–55] and diameters [54] were chosen as representative of an average adult femoral artery. The lengths of each artery were chosen such that blood flow would establish a full parabolic profile prior to reaching the subsequent bifurcation. The inlet velocity boundary condition has a parabolic profile along the parent artery as given by:

$$V=V_{\max}(1-\frac{r^{2}}{R^{2}})$$ (9)

where *V* is the velocity at radial distance *r* from the center line of the artery and *R* is the artery radius. At the outlets of the idealized femoral artery tree, traction-free boundary condition is applied. The no-slip boundary condition was prescribed along all artery walls. Transient numerical simulations were performed across two inflow waveforms (two seconds), and numerical results presented in this paper were extracted from the second cycle to minimize effects of the initial flow condition. The radius of curvature (R_c_) chosen will lead to pressure spikes at the bifurcation points of the idealized femoral artery tree. Using a larger R_c_ would distribute pressure more evenly across the bifurcation, however this smaller R_c_ was chosen for two reasons. First, it shows that our numerical method is stable and achieves convergence even for tougher numerical problems. Second, it is biologically relevant for stress to accumulate at artery bifurcations, which is the case for aneurysm development [56]. Grid convergence tests were performed on this artery to ensure that converged numerical results were obtained with mesh refinement (S1 Fig). Numerical parameters used in the simulations on the idealized femoral artery tree, as well as other geometric configurations are also provided (S1 Table).

### Atherosclerosis simulations

Atherosclerotic lesions were added to the idealized femoral artery tree in one of two regions: within the parent artery or within the first bifurcation. Atherosclerotic lesions within the parent artery were numerically modeled for three different degrees of blocking: namely, 25%, 50%, and 75% of the parent artery. Lesions within the first bifurcation blocked 35%, 50%, or 72% of the daughter artery.

*Software*. All geometries were developed in Autodesk Inventor and meshes were created using Abaqus/CAE. Atherosclerosis was modeled as a rigid wall with no slip conditions. Numerical tests were carried out with our in-house code that is based on the method presented here. Post-processing and visualization of numerical data was carried out with Paraview.

## Results

### Idealized Bifurcating Artery Geometries

**Fig 2:**
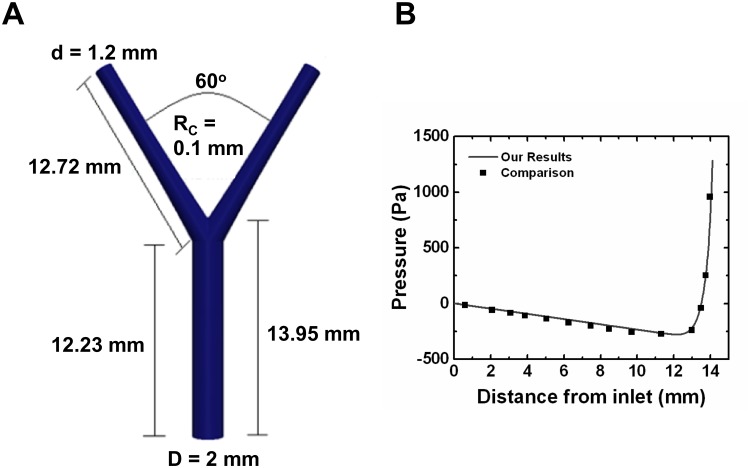
Numerical Method Verification via a 3-Dimensional Idealized Bifurcating Artery. (A) The 3-dimensional idealized bifurcating artery proposed by Chung [45] was used for verifying our method. The parent artery where the inflow is prescribed is 2 mm in diameter, 12.23 mm in length up to the bifurcation, and 13.95 mm in length to the bifurcation apex. The daughter arteries are both 1.2 mm in diameter and 12.72 mm in length from the bifurcation. The radius of curvature (R_c_) is 0.1 mm, and the angle between the daughter artery walls is 60°. (B) Pressure was extracted along the center of the parent artery from the inflow to the apex of the bifurcation.

Our numerical method shows excellent agreement with the idealized single bifurcating artery simulations by Chung (Fig 2A) [45]. For this verification study, the Newtonian model was employed with viscosity = 0.00345 Pa/s, density = 1050 kg/m^3^, and *Re* = 505. Pressure along the center of the parent artery from the inlet to the apex was compared for the verification (Fig 2B).

### Velocity profiles throughout the idealized femoral artery tree

**Fig 3:**
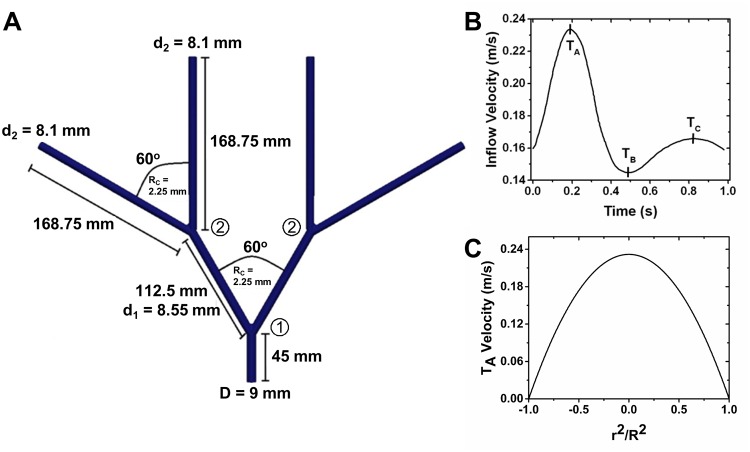
3-Dimensional Idealized Femoral Artery Tree and Inflow Conditions. (A) Dimensions of the idealized femoral artery tree. The bifurcations are defined by the circled numbers, with the first bifurcation defined as the 9 mm artery bifurcating into 8.55 mm arteries and the second bifurcation defined as an 8.55 mm artery bifurcating into 8.1 mm arteries. In both bifurcations, centerline to centerline of the daughter arteries = 60° and the R_c_ = 2.25 mm. Dimensions for only one side of the idealized femoral artery tree are shown due to symmetry. (B) The inflow velocity waveform applied at the geometric center of the parent artery. Marked peaks on the inflow velocity waveform are time points where comparisons of the two models are drawn. (C) Example inlet boundary condition across the parent artery at T_A_. X-axis is shown as squared distance from the vessel center (r) over the squared vessel radius (R). The origin (0,0,0) of the idealized femoral artery tree is defined as the geometric center of the parent artery, and all spatial locations given for this geometry are based on this origin in Cartesian coordinates.

**Fig 4:**
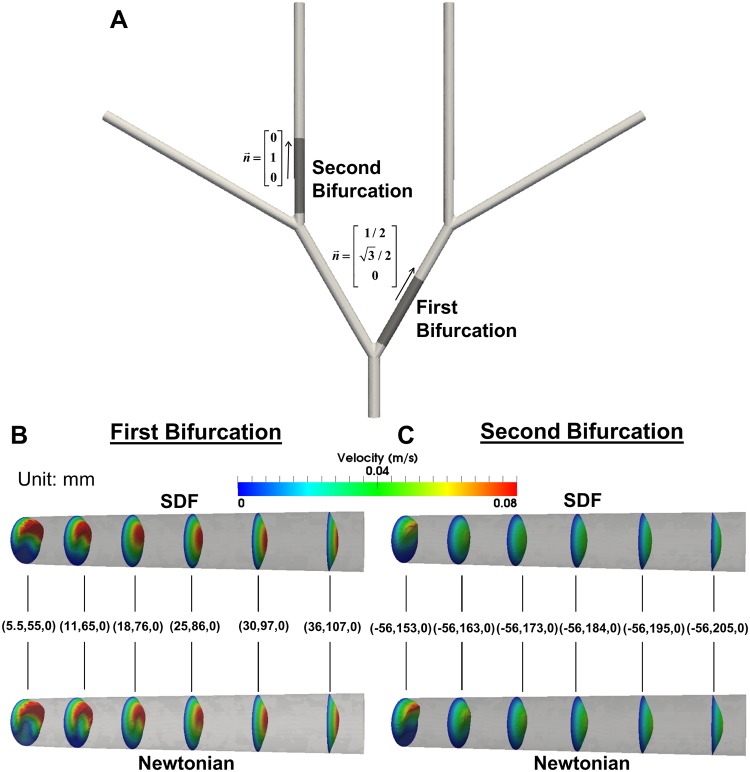
Daughter Artery Velocity Profiles. Daughter artery velocity profiles immediately following the idealized femoral artery tree bifurcations at T_B_. (A) Locations along the idealized femoral artery tree where the contours were extracted. Velocity profiles in daughter arteries following (B) the first bifurcation and (C) the second bifurcation. Artery walls adjacent to bifurcations are portrayed as the top wall, and artery walls opposite bifurcations are portrayed as the bottom wall. Velocity slices were extracted from planes intersecting the idealized femoral artery tree. The (x,y,z) coordinates in mm are given for each velocity slice indicates the origin of the plane, which were oriented perpendicular to the normal vectors ($\vec{n}$) given for each daughter artery.

To investigate hemodynamic forces within a more relevant physiology, we developed an idealized femoral artery tree (Fig 3A). Three time points were chosen as the comparison points between models, as defined by T_A_, T_B_, T_C_ (Fig 3B). A sample inflow velocity waveform at T_A_ is given (Fig 3C). Velocity contours at T_B_ were extracted in daughter arteries immediately following the first and second bifurcation (Fig 4A). The Newtonian model requires longer artery distance to establish a parabolic profile following the first (Fig 4B) and second bifurcations (Fig 4C) than the SDF model. Additionally, the Newtonian model gives lower velocities near the artery wall opposite the bifurcation point (Fig 4B and 4C).

**Fig 5:**
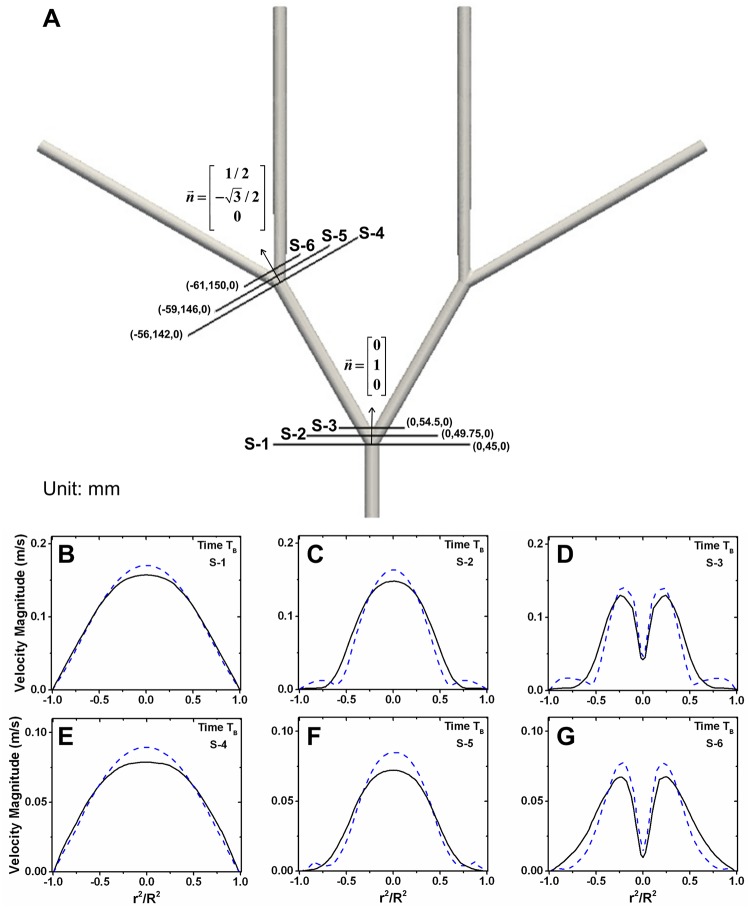
Velocity Profiles within the Idealized Femoral Artery Tree Bifurcations at T_B_. (A) Velocity profiles plotted over the cross sections shown at T_B_. Velocity slices across first bifurcation were extracted at (B) the bifurcation start, (C) bifurcation midpoint, and (D) the bifurcation apex. Velocity slices were extracted at respective locations (E-G) across the second bifurcation. X-axis is shown as squared distance from the vessel center (r) over the squared vessel radius (R). For slices within the bifurcations (C, D, F, G) the vessel is not cylindrical, so R is chosen as half the wall to wall length. The solid black line is the SDF velocity profile and the dashed blue line is the Newtonian velocity profile. Velocity profiles were extracted from slices originated at the (x,y,z) coordinates in mm given, and slices were oriented perpendicular to the normal vectors ($\vec{n}$) given for each bifurcation.

Velocity profiles across cross sections of the first and second bifurcations were extracted at T_B_ (Fig 5A). At the beginning of bifurcations the SDF velocity profiles exhibit a flatter peak than the Newtonian velocity profiles (Fig 5B, 5E). The SDF model undergoes less fluid reversal near the artery walls within the bifurcation than the Newtonian model (Fig 5C, 5D and 5F, 5G). The SDF model peak velocity is also lower than the Newtonian peak velocity across every slice (Fig 5B–5G). For verification purposes, velocity profile slices at T_A_ and T_C_ are also given (S2, S3 Figs).

**Fig 6:**
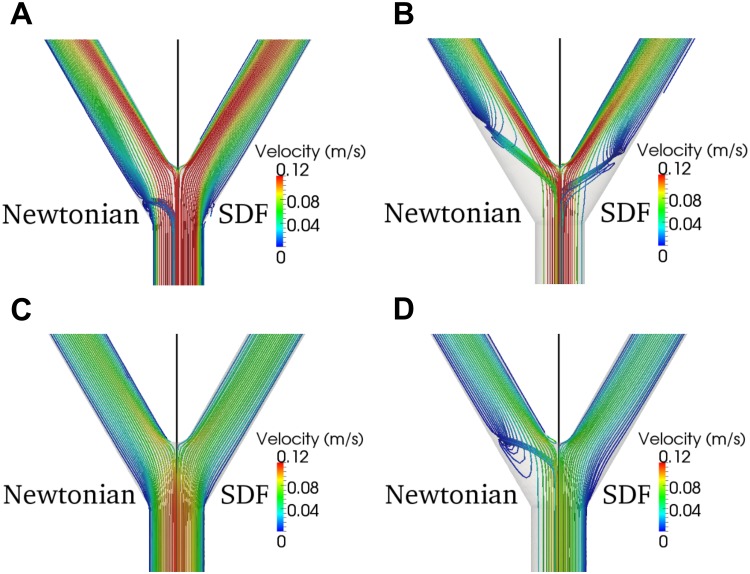
Velocity Streamlines through the Idealized Femoral Artery Tree Bifurcations. Velocity streamlines through the idealized femoral artery tree bifurcations for both models. (A) The first bifurcation at T_A_, (B) the first bifurcation at T_B_, (C) the second bifurcation at T_A_, and (D) the second bifurcation at T_B_. Due to symmetry, only one half of the idealized femoral artery tree is shown. The left half is the Newtonian model and the right half is the SDF model, separated across the black line.

Velocity streamlines show that at T_A_ there is no noticeable difference in velocity between the two models at both the first (Fig 6A) and second (Fig 6C) bifurcations. At T_B_ both models exhibit noticeable fluid reversal following the first bifurcation, but the SDF fluid reversal is less pronounced than the Newtonian fluid reversal (Fig 6B). While the Newtonian model still exhibits fluid reversal within the second bifurcation, no fluid reversal is noticeable with the SDF model (Fig 6D).

### Pressure drop across the idealized femoral artery tree

**Fig 7:**
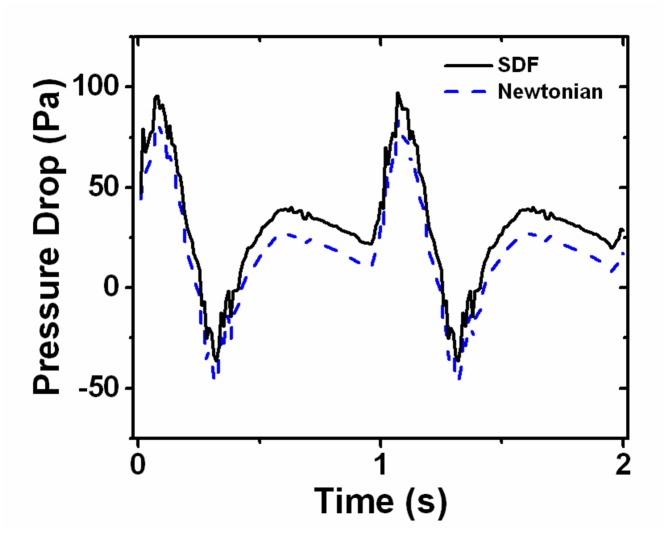
The Pressure Drop Across the Idealized Femoral Artery Tree. The pressure drop (P_inflow_—P_outflow_) across the idealized femoral artery tree given by both models across two inflow waveforms. Due to symmetry, the pressure at all four outlets is identical, and thus any outlet artery can be chosen.

The SDF model always gives an equal or higher pressure drop (inlet pressure minus outlet pressure) across the idealized femoral artery tree than the Newtonian model (Fig 7). The largest difference in pressure drop occurs at T_B_, where the Newtonian model gives 42.8% the pressure drop given by the SDF model (Table 1). The pressure drop given by the two models is most similar at T_A_, where the Newtonian model gives 76.5% the pressure drop given by the SDF model (Table 1).

**Table 1:** The pressure drop across the idealized femoral artery tree.

	Time T_A_	Time T_B_	Time T_C_
Newtonian (Pa)	42.26	8.42	21.39
SDF (Pa)	55.25	19.66	33.29
Percent (NSE/SDF)	76.5	42.8	64.2

The pressure drop (P_inflow_—P_outflow_) given by the two models at T_A_, T_B_, and T_C_. The percent is the pressure drop given by the Newtonian model divided by the pressure drop given by the SDF model.

#### Remark 1

In fluid dynamics governed by the incompressible momentum balance equations, the pressure field is computed up to an arbitrary constant. However the pressure drop computed by the momentum balance equations is accurate. In engineering systems where the value of the pressure field is invariably known at some point in the domain, prescribing that known value as a boundary condition eliminates the arbitrary constant from the pressure field. In the present context of blood flow through arteries, a precise value of the pressure field to be applied as a pressure boundary condition is not readily available. Consequently, we have opted for traction-free boundary conditions on the outlet, making the average value of the outlet pressure equal to zero. It is important to note that even in this case the pressure drop between the inflow and outflow is accurately captured by the model.

### WSS across the idealized femoral artery tree

**Fig 8:**
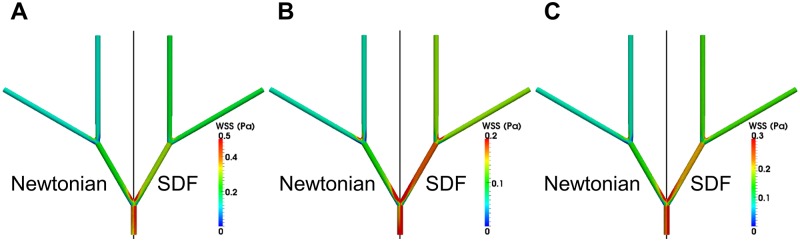
WSS Contours across the Idealized Femoral Artery Tree. WSS contours at instantaneous time points (A) T_A_, (B) T_B_, and (C) T_C_ the idealized femoral artery tree. Due to symmetry, only one half of the idealized femoral artery tree is shown for each model, where the left half is the Newtonian model and the right half is the SDF model, separated across the black line.

WSS contours show that at T_A_ the SDF model gives noticeably higher WSS along both sets of daughter arteries than the Newtonian model (Fig 8A). WSS is computed by:

WSS=(σ⋅n)−((σ⋅n)⋅n)n (10)

where σ is the stress tensor and **n** is the surface normal vector. The difference between SDF and Newtonian WSS across the entire idealized femoral artery tree is qualitatively most noticeable at T_B_ (Fig 8B). At T_C_, there is a larger difference in WSS at the bifurcation points between the two models than at the other two time points (Fig 8C).

**Fig 9:**
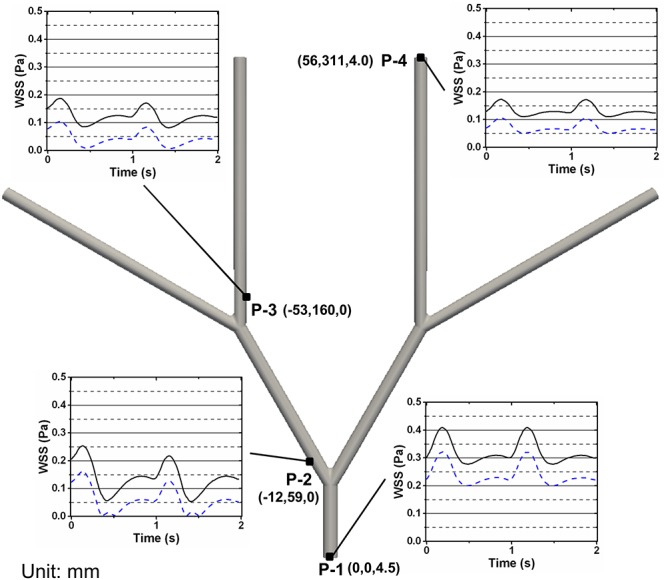
WSS Versus Time Across the Idealized Femoral Artery Tree. WSS versus time at different spatial locations on the idealized femoral artery tree given by the (x,y,z) coordinates in mm. WSS is shown at the inflow wall (P-1), the wall opposite the first bifurcation (P-2), the wall opposite the second bifurcation (P-3), and the outflow wall (P-4). The solid black line is the SDF WSS and the blue dashed line is the Newtonian WSS.

The SDF model always gives higher WSS compared to the Newtonian model at the inflow, outflow, and outer bifurcation walls along the idealized femoral artery tree (Fig 9). At the inflow wall, the Newtonian model gives a mean WSS of 0.24 Pa and the SDF model gives a mean WSS of 0.32 Pa (Fig 9P-1). At the wall opposite the first bifurcation, the Newtonian model gives a mean WSS of 0.06 Pa and the SDF model gives a mean WSS of 0.14 Pa (Fig 9P-2). At the wall opposite the second bifurcation, the Newtonian model gives a mean WSS of 0.04 Pa and the SDF model gives a mean WSS of 0.12 Pa (Fig 9P-3). At the outflow wall, the Newtonian model gives a mean WSS of 0.07 Pa and the SDF model gives a mean WSS of 0.13 Pa (Fig 9P-4). Thus, the mean WSS of the two models was most similar at the inflow wall and least similar at the wall opposite the second bifurcation.

**Fig 10:**
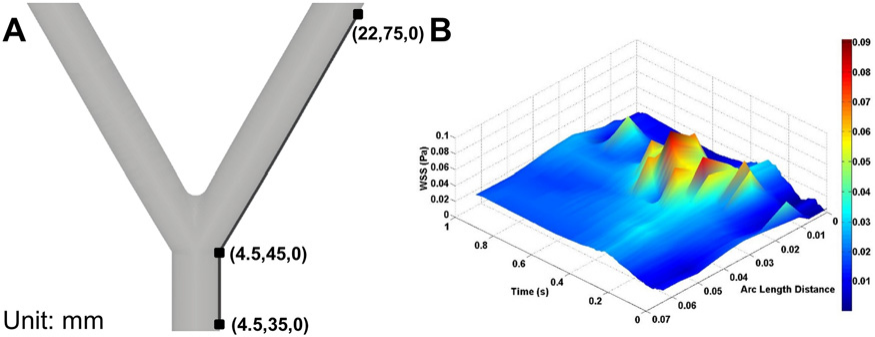
WSS Across the Wall Opposite the First Bifurcation Versus Time and Spatial Location. (A) WSS was extracted along the wall opposite the first bifurcation shown by the black line. The (x,y,z) coordinates in mm are given for the three points shown. (B) The WSS magnitude difference between the two models (|SDF_WSS_−Newtonian_WSS_|) versus time and spatial location. Spatial location is given as arc length distance, where arc length of zero corresponds to the lowest spatial location along the artery wall.

WSS along the wall opposite the first bifurcation was extracted as a function of spatial location and time (Fig 10A). The difference in WSS between the two models (|SDF_WSS_−Newtonian_WSS_|) shows that the largest difference in WSS occurs at the start of the bifurcation (0.03 arc length) at T_B_ (0.5s) (Fig 10B). Following the bifurcation, the WSS difference holds approximately constant at 0.02 Pa for every time and spatial location (Fig 10B).

### Comparison of SDF and Newtonian models across an atherosclerotic artery

**Fig 11:**
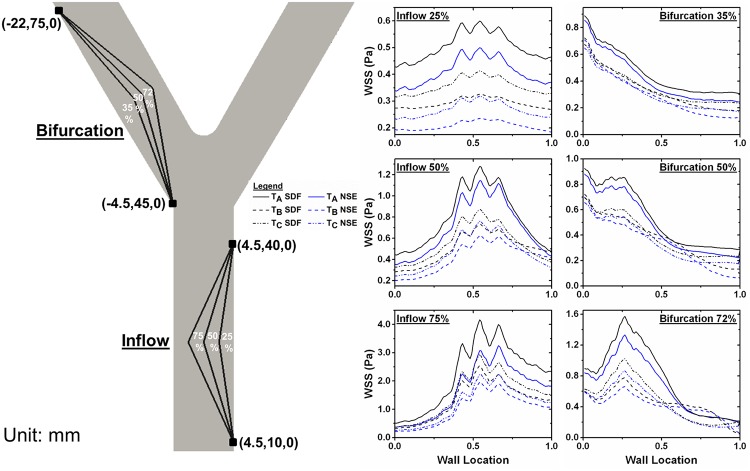
WSS at Varying Degrees of Atherosclerosis. Atherosclerotic lesions of varying degrees were induced in the idealized femoral artery tree at the inflow artery or at the first bifurcation. The (x,y,z) coordinates in mm show were the atherosclerotic lesion endpoints were prescribed. WSS from the SDF and Newtonian models were extracted along the wall opposite the plaque and plotted at T_A_, T_B_, and T_C_. The x-axis indicates location along the wall, where zero corresponds to the lowest y-coordinate of the plaque and one as the highest y-coordinate.

WSS given with the SDF model is higher than WSS with the Newtonian model for every degree of atherosclerosis simulated (Fig 11). As the degree of atherosclerosis increases, WSS along the opposite wall also increases; the maximum WSS in parent artery atherosclerosis at T_A_ with the SDF model is 0.60 Pa at 25% artery blockage, 1.28 Pa at 50% blockage, and 4.17 Pa at 75% blockage. Conversely, the Newtonian model gives 0.50 Pa at 25% artery blockage, 1.15 Pa at 50% blockage, and 3.25 Pa at 75% blockage. The maximum WSS in bifurcation atherosclerosis at T_A_ with the SDF model is 0.89 Pa at 35% artery blockage, 0.93 Pa at 50% blockage, and 1.58 Pa at 72% blockage. Conversely, the Newtonian model gives 0.86 Pa at 35% artery blockage, 0.88 Pa at 50% blockage, and 1.33 Pa at 72% blockage.

## Discussion

We compared blood velocity, pressure, and WSS given by SDF and Newtonian models. We confirm that differences between models in these comparisons do not arise from numerical error by verifying our stabilized finite element method with the 2-dimensional lid-driven cavity flow problem and the idealized single bifurcating artery. Our numerical simulations show that the Newtonian velocity profiles scale linearly with cavity height and width dimensions, while the SDF velocity profiles scale nonlinearly. In the 3-dimensional lid-driven cavity flow, the SDF model gives equivalent or larger WSS and wall pressure than the Newtonian model across the entirety of the cavity walls. Within the idealized femoral artery tree, the SDF model gives lower peak blood velocities, yet higher pressure drops, than the Newtonian model. Our numerical simulations show that the Newtonian model exhibits less WSS than the SDF model at every time and spatial location along the idealized femoral artery tree under both healthy and atherosclerotic conditions. These differences are attributed solely to the difference in blood viscosity between the two models; the Newtonian blood viscosity is constant whereas the SDF blood viscosity is dependent on the blood shear-rate.

### Newtonian velocity profiles scale linearly with cavity size

The Newtonian model gives similar velocity profiles through the 2-dimensional cavity scaled at differential height and width dimensions, given that *Re* is held constant. Conversely, the SDF velocity profiles scale nonlinearly with cavity height and width. This occurs because cavity scaling alters shear-rate magnitude and distribution throughout the cavity, which does not affect Newtonian velocity profiles as the Newtonian viscosity is solely determined by *Re*. This result implies Newtonian velocity profiles through identical arteries at different scales will be identical, but this is not physiologically relevant due to the shear-rate dependent viscosity of blood. This physiological irrelevancy could alter the predicted distribution of flowing platelets throughout a blood vessel, which is controlled by the blood velocity profile and important in wound healing and atherosclerotic lesion formation, [57]. Thus, numerical simulations using Newtonian blood models can provide inaccurate velocity profiles resulting in incorrect risk estimations of hemodynamic-dependent diseases.

### The Newtonian model gives higher peak velocities throughout the idealized femoral artery tree than the SDF model

Our numerical simulations show that the Newtonian peak velocity through the parent artery of the idealized femoral artery tree is 0.075 m/s greater than the SDF peak velocity. Physiologically relevant peak blood velocities are critical for risk estimation of scleroderma and systemic sclerosis development in response to abnormally low blood velocities [58]. Magnetic nanoparticles administered to disrupt atherosclerotic lesion formation are also highly dependent on peak blood velocities [59, 60]. Peak blood velocities provided by numerical simulations must be physiologically relevant in order to accurately estimate hemodynamic-dependent disease progression.

### The Newtonian model gives lower pressure drops across the idealized femoral artery tree than the SDF model

Our numerical simulations show that the pressure drop across the idealized femoral artery tree with the SDF model is approximately 2.3-fold higher than that with the Newtonian model. Also, the SDF pressure drop across the 3-dimensional cavity is approximately 5-fold higher than the Newtonian pressure drop. These differences are significant because abnormally low pressure drops occur over flow resistive arterial regions which have high likelihood of stenosis formation [61]. Thus, it is essential that numerical simulations provide physiologically relevant pressure maps to accurately predict hemodynamic-dependent disease progression.

### Comparison of numerically predicted WSS to empirical measurements

The Newtonian model gives a mean WSS of 0.24 Pa along the parent artery of the idealized femoral artery tree whereas the SDF model gives a mean WSS of 0.32 Pa. Due to the prescribed dimensions and inflow waveform, the parent artery corresponds to the common femoral artery. Ultrasound measurements found WSS in the common femoral artery to be 0.35 ± 0.03 Pa in one study [62] and 0.36 ± 0.02 Pa in another [63] (mean ± standard error of the mean). Thus, WSS given by the SDF model matches well with empirical data, whereas WSS given by the Newtonian model does not match empirical data as effectively.

### The Newtonian model exhibits less WSS than the SDF model across the idealized femoral artery tree in disease-free physiology

This difference in WSS of 0.12 Pa between the Newtonian and SDF models in healthy physiology is significant and will result in differential predictions of physiological processes. For example, laminar flow chamber experiments correlate this difference with a 3-fold higher amount of monocytes adhering to endothelial cells predicted by the Newtonian model than the SDF model; monocyte adhesion to the vascular wall contributes to atherosclerotic lesion development and rupture [64]. This WSS difference between models is also significant for predicting growth of atherosclerotic lesions or aneurysms [65–68] and vascular remodeling through integrin, adhesion molecule, and ion channel mechanotransduction [69–71]. For example, atherosclerotic lesion growth studies measured that arteries experiencing 0.12 less WSS had an increased lesion growth of 0.05 mm over the course of a year [66]. Patch clamp experiments on endothelial cells measured a K^+^ current of approximately 21 pA when a WSS of 0.086 Pa was applied, versus a K^+^ current of approximately 5 pA when a WSS of 0.035 Pa was applied [71]. This 4-fold increase in K^+^ current given a WSS difference of 0.05 Pa indicates that the difference of 0.12 Pa between the two models will result in significantly different endothelial cell K^+^ current predictions. These studies coupled with our numerical simulations imply that physiologically relevant WSS in disease-free models is essential to accurately predict induction of hemodynamic-dependent diseases.

### The Newtonian model gives less WSS than the SDF model across the idealized femoral artery tree in atherosclerotic conditions

Our numerical simulations show that the SDF model gives up to 1.09 Pa greater WSS than the Newtonian model across multiple degrees of atherosclerotic lesion progression in the idealized femoral artery tree. This difference is significant because the number of monocyte to endothelial cell adhesions, important to atherosclerotic lesion development and rupture, increased approximately 3-fold after decreasing WSS by 0.1 Pa (1 dyn/cm^2^) in laminar flow chamber experiments [64]. Higher WSS also increases multimeric von Willebrand factor uncoiling, which is localized to injured vessel walls to adhere and activate platelets to facilitate clotting and wound healing [72–75]. Thus, physiologically relevant WSS provided by numerical simulations in atherosclerotic conditions is essential to properly assess risk of further atherosclerotic lesion progression.

### Limitations of idealized femoral artery tree numerical simulations

The idealized femoral artery tree consists of a rigid body, whereas arteries physiologically exhibit elastic properties. Previous studies have sought to determine the significance of compliant artery walls compared to rigid walls. Leung *et al* found a negligible difference of less than 1% in the peak WSS determined by the compliant and rigid wall models using patient-specific abdominal aortic aneurysm geometries [76]. However, Scotti *et al* found that in abdominal aortic aneurysms with varying wall thickness and asymmetry the rigid wall model underestimated WSS by up to 30.2% [77]. In atherosclerotic carotid arteries, Yang *et al* found that differences in maximum shear-stress between rigid and compliant artery wall models were significant within flow-stagnation regions, but insignificant within high shear-stress regions [19]. These discrepancies indicate a need to better understand under what circumstances compliant artery wall models are necessary. The idealized femoral artery tree can serve as a test bed for identifying the conditions under which a coupled blood-artery wall model would be necessary.

### Future directions

Our ability to numerically simulate transient blood flow can be applied to predict the efficacy of exercise regimes in preventing or treating diseases. For example, exercise is highly beneficial in the prevention of Type 2 diabetes [78, 79], but the type and rigor a diabetic patient should undergo is not well defined [80]. Exercise also remains the best intervention for peripheral artery disease through its induction of angiogenesis leading to an increased capillary density [81]. Numerical simulations could optimize exercise regimes for individual patients. However, properly optimized exercise regimes require accurate arterial wall properties. For example, exercise in healthy patients was found to be associated with a 50% decrease in pulmonary artery resistance but a 30% increase in pulmonary artery compliance [82], indicating that artery resistance and compliance are inversely correlated [83]. Age and sex can also be an important factor in artery wall properties, as women and patients under 50 years old were shown to have greater distensibility in pulmonary arteries [84]. These changes in artery wall properties are likely dependent in part on the increased cardiac output and artery pressures observed in exercise [85]. Thus, as our current numerical method is limited to modeling arterial walls as rigid bodies, extending this numerical method to account for arterial compliance and resistance changes is necessary to predict optimized exercise regimes. Hemodynamic models can also be applied towards developing and optimizing therapeutic approaches. For example, tumor blood flow can be constricted through rupturing microvessel with microbubbles [86], and numerical simulations could predict which microvessels should be targeted to most effectively starve tumors. Extending hemodynamic models to dynamically model physiological processes would provide additional insight into the process. For example, previous studies dynamically modeling sprouting angiogenesis revealed the dependency of molecular concentrations to blood flow [87, 88]. In all such studies, modeling hemodynamic forces and molecular interactions would provide a comprehensive overview of the biological process, allowing novel insight to be drawn or therapy optimizations to be developed.

### Concluding remarks

We present a 3-dimensional idealized femoral artery tree that can be used as a test bed for hemodynamic simulations through an easily reproducible geometry. We show that Newtonian models of blood yield lower WSS than the SDF model throughout the idealized femoral artery tree in both healthy and atherosclerotic conditions. Newtonian velocity profiles are shown to exhibit higher peak magnitudes than the SDF model within the healthy idealized femoral artery tree. Newtonian pressure is shown to be lower than SDF pressure within the 3-dimensional lid-driven cavity flow and the idealized femoral artery tree. Additionally, Newtonian velocity profiles scale linearly with the 2-dimensional height and width, whereas SDF velocity profiles scale nonlinearly. These differences are attributed to the non-Newtonian constitutive relation in the SDF model that is based on the empirically observed rheology of blood. We provide quantitative analysis of velocity, WSS, and pressure throughout the idealized femoral artery tree that can be used as a benchmark for future studies. Future work will extend these ideas into patient-specific geometries or integrate non-Newtonian hemodynamics with molecular signaling to model atherosclerosis development.

## Supporting Information

S1 Fig Grid convergence tests on the idealized femoral artery tree at Re = 550. Grid convergence tests were performed using (A) the velocity profile across the first bifurcation (slice at y = 54.2 mm), (B) the velocity profile across the second bifurcation (slice at y = 160 mm after rotating the idealized femoral artery tree by 30° in the z-direction), and (C) WSS at the four points along the idealized femoral artery tree as given in Fig 9. Graph inserts are a zoomed in comparison between the meshes near the center of the bifurcation. Five meshes were used for the convergence test, with the first figure legend indicating the approximate number of elements each mesh contains. The mesh used for simulations in this study contains approximately 173,000 elements. Note that grid convergence tests were not performed for the 2-dimensional cavity or the single bifurcating artery as validations were provided for those geometries. For the 3-dimensional cavity geometry, our group provided convergence tests in a previous manuscript [33].”
(TIF)

S2 Fig Velocity Profiles within the Idealized Femoral Artery Tree Bifurcations at T_A_. (A) Velocity profiles plotted over the cross sections shown at T_A_. Velocity slices across first bifurcation were extracted at (B) the bifurcation start, (C) bifurcation midpoint, and (D) the bifurcation apex. Velocity slices were extracted at respective locations (E-G) across the second bifurcation. X-axis is shown as squared distance from the vessel center (r) over the squared vessel radius (R). For slices within the bifurcations (C, D, F, G) the vessel is not cylindrical, so R is chosen as half the wall to wall length. The solid black line is the SDF velocity profile and the dashed blue line is the Newtonian velocity profile.
(TIF)

S3 Fig Velocity Profiles within the Idealized Femoral Artery Tree Bifurcations at T_C_. (A) Velocity profiles plotted over the cross sections shown at T_C_. Velocity slices across first bifurcation were extracted at (B) the bifurcation start, (C) bifurcation midpoint, and (D) the bifurcation apex. Velocity slices were extracted at respective locations (E-G) across the second bifurcation. X-axis is shown as squared distance from the vessel center (r) over the squared vessel radius (R). For slices within the bifurcations (C, D, F, G) the vessel is not cylindrical, so R is chosen as half the wall to wall length. The solid black line is the SDF velocity profile and the dashed blue line is the Newtonian velocity profile.
(TIF)

S1 File Newtonian and SDF fluid model comparison in the lid-driven cavity flow problem. Lid-driven flow was applied to 2-dimensional and 3-dimensional cavities, and velocity, pressure, and WSS profiles were compared between the Newtonian and SDF fluid models. Fig A, Numerical Method Verification with the 2-Dimensional Lid-Driven Cavity Flow Problem. Verification of our numerical method using simulations provided by Ghia in the 2-dimensional lid-driven cavity flow problem [44]. (A) Flow profile throughout the cavity from the Newtonian model at *Re* = 10,000. V_x_ was extracted along the white line in the geometric center of the cavity at (A) *Re* = 1,000; (B) *Re* = 5,000; and (C) *Re* = 10,000. The bottom of the cavity is defined as y = 0 and the top as y = 1. Fig B, Effect of Cavity Size and *Re* on Newtonian Velocity Profiles. Effect of the cavity size on the Newtonian velocity profiles across the geometric center of the 2-dimensional cavity at (A) *Re* = 500, (B) *Re* = 5,000, and (C) *Re* = 10,000. The full cavity size is 1m x 1m, the 10% size is 0.1 m x 0.1 m, and the 1% size is 0.01 m x 0.01 m. The y-coordinate of the smaller cavities are scaled up for visualization purposes. Fig C, Effect of Cavity Size and *Re* on SDF Velocity Profiles. Effect of the cavity size on the SDF velocity profiles across the geometric center of the 2-dimensional cavity at (A) *Re* = 500, (B) *Re* = 5,000, and (C) *Re* = 10,000. The full cavity size is 1m x 1m, the 10% size is 0.1 m x 0.1 m, and the 1% size is 0.01 m x 0.01 m. The y-coordinate of the smaller cavities are scaled up for visualization purposes. Fig D, Pressure and Velocity Profiles in the 3-Dimensional Cavity. (A) The 3-dimensional cavity (1m x 1m x 1m) with inflow applied across the top of the cavity in the positive x-direction. (B) Pressure and (C) V_x_ profiles were extracted along the red line parallel to the y-axis shown. The bottom of the cavity is defined as y = 0 and the top of the cavity as y = 1. Fig E, WSS and Wall Pressure along the 3-Dimensional Cavity. (A) WSS across the 3-dimensional cavity for the SDF (left) and Newtonian (right) models. (B) The 3-dimensional cavity where (C) WSS and (D) wall pressure were extracted along the red line parallel to the y-axis and along the right wall. The bottom of the cavity is defined as y = 0 and the top of the cavity is y = 1. Table A, The lowest V_x_ magnitude for the 2-dimensional cavity at different sizes. The lowest V_x_ magnitude in mm/s along the geometric center of the 2-dimensional cavity for the Newtonian and SDF models. The bottom of the cavity is defined as y = 0 m and the top as y = 1 m. Only one cavity size is shown for the Newtonian model. Table B, Spatial locations of kinks in V_x_ profiles in the 2-dimensional cavity at different sizes. The location of kinks in the V_x_ profile from the bottom of the cavity along the geometric center for the Newtonian and SDF models. Kinks are seperated by a semi-colon for that particular *Re*; if only one kink is present the second is labeled as “-“. Only one cavity size is shown for the Newtonian model. Table C, Velocity and pressure differences between the two models in the 3-dimensional cavity. The lowest V_x_ magnitude and spatial location, lowest pressure magnitude and spatial location, and highest press magnitude for the Newtonian and SDF models across the geometric center of the 3-dimensional cavity at *Re* = 1,000. Spatial location are given in distance from the bottom of the cavity along the geometric center of the cavity. Highest pressure spatial location is always located at the top of the cavity, and is thus omitted.
(DOCX)

S1 Table Numerical parameters for simulations of each geometry used in this study. The element type used was either the 8-node hexahedral (HEX8) or the 10-node tetrahedral (TET10) element. Note that the 2-dimensional cavity was created as a 3-dimensional geometry using HEX8 elements with the cavity thickness (z-dimension) being 20% of the height and width (0.05 m for the full size cavity). However, the z-directional velocity was held constant at zero to produce 2-dimensional physics from a 3-dimensional geometry. We conducted the 2-dimensional cavity simulations in this way because our numerical method provides greater stability and convergence rates for 3-dimensional element types. All geometries except for the idealized femoral artery tree were simulated at steady state, and thus, given the chosen initial velocity, the number of time steps taken were used to reach the desired *Re*. The idealized femoral artery tree was simulated transiently, and 200 time steps were chosen to simulate the time course of 2 seconds. The *Re* indicated for the idealized femoral artery tree is the mean *Re* through the parent artery over the inflow velocity waveform.
(DOCX)
