# Supplementary figures and images for: Hemodynamic Analysis in an Idealized Artery Tree: Differences in Wall Shear Stress between Newtonian and Non-Newtonian Blood Models
Source: PLoS One. 2015 Apr 21;10(4):e0124575. doi: 10.1371/journal.pone.0124575 (PMC4405589; doi:10.1371/journal.pone.0124575)

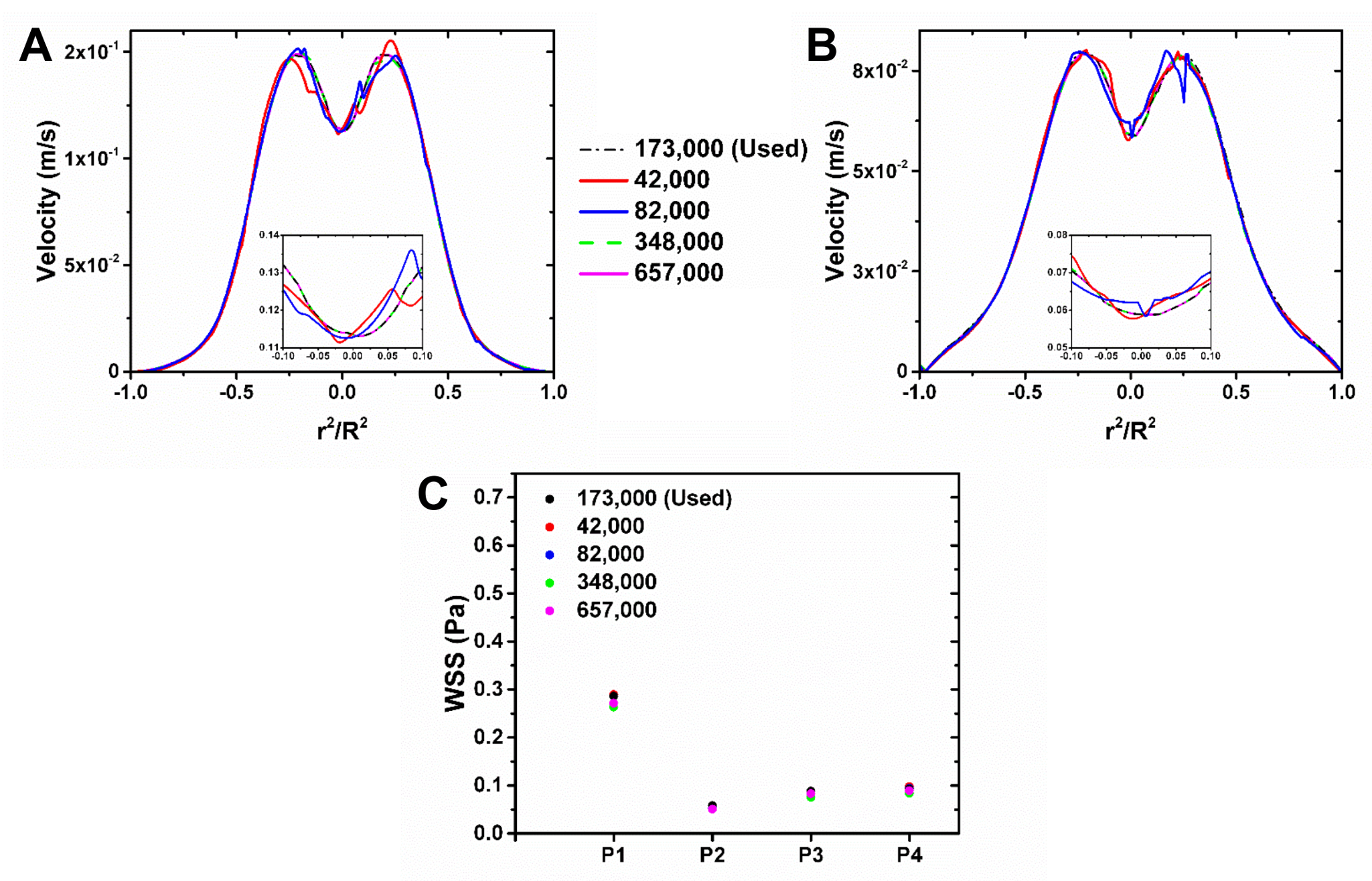

Supplement: S1 Fig — Grid convergence tests were performed using (A) the velocity profile across the first bifurcation (slice at y = 54.2 mm), (B) the velocity profile across the second bifurcation (slice at y = 160 mm after rotating the idealized femoral artery tree by 30° in the z-direction), and (C) WSS at the four points along the idealized femoral artery tree as given in Fig 9. Graph inserts are a zoomed in comparison between the meshes near the center of the bifurcation. Five meshes were used for the convergence test, with the first figure legend indicating the approximate number of elements each mesh contains. The mesh used for simulations in this study contains approximately 173,000 elements. Note that grid convergence tests were not performed for the 2-dimensional cavity or the single bifurcating artery as validations were provided for those geometries. For the 3-dimensional cavity geometry, our group provided convergence tests in a previous manuscript [33].” (TIF) [file pone.0124575.s001.tif]

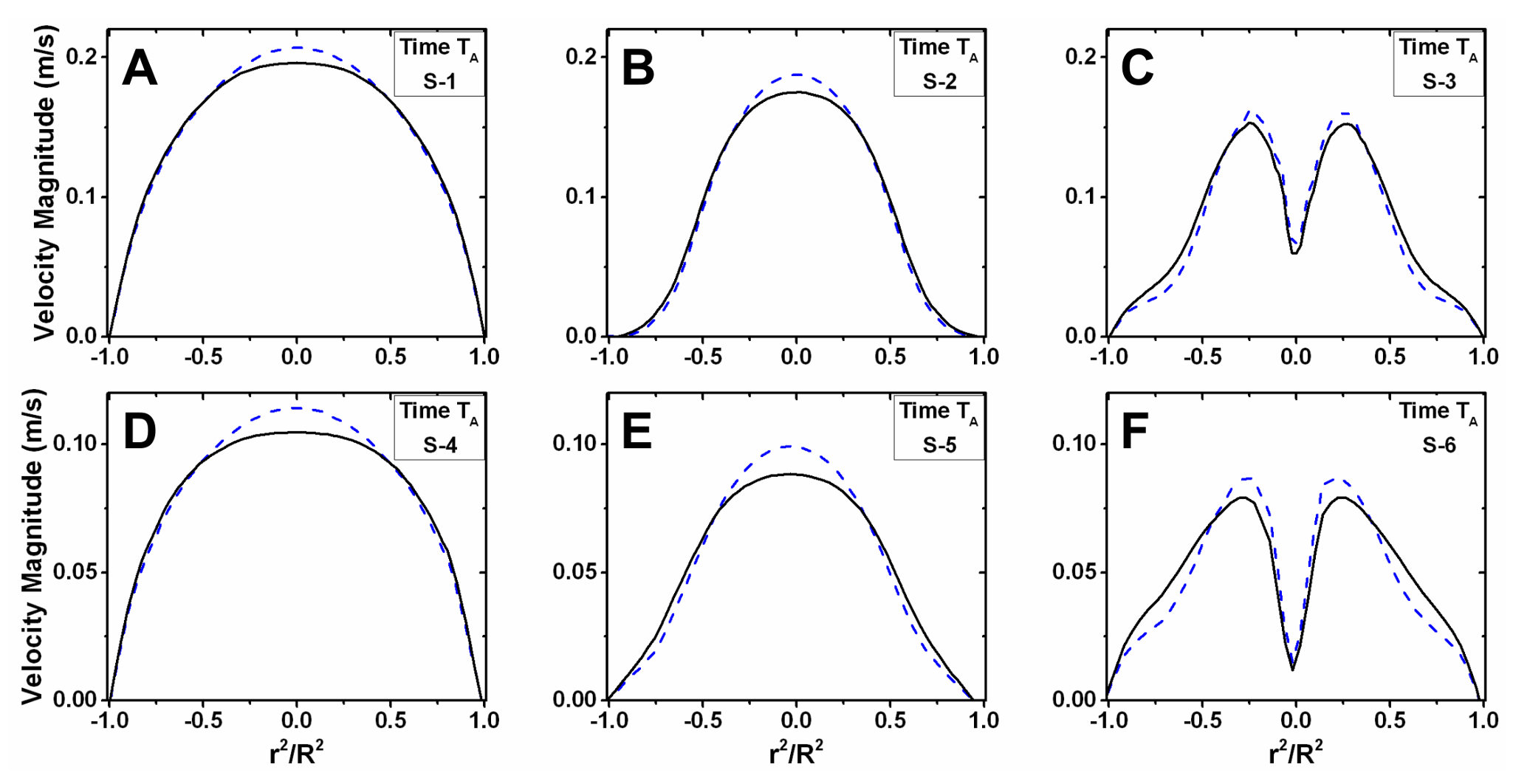

Supplement: S2 Fig — (A) Velocity profiles plotted over the cross sections shown at TA. Velocity slices across first bifurcation were extracted at (B) the bifurcation start, (C) bifurcation midpoint, and (D) the bifurcation apex. Velocity slices were extracted at respective locations (E-G) across the second bifurcation. X-axis is shown as squared distance from the vessel center (r) over the squared vessel radius (R). For slices within the bifurcations (C, D, F, G) the vessel is not cylindrical, so R is chosen as half the wall to wall length. The solid black line is the SDF velocity profile and the dashed blue line is the Newtonian velocity profile. (TIF) [file pone.0124575.s002.tif]

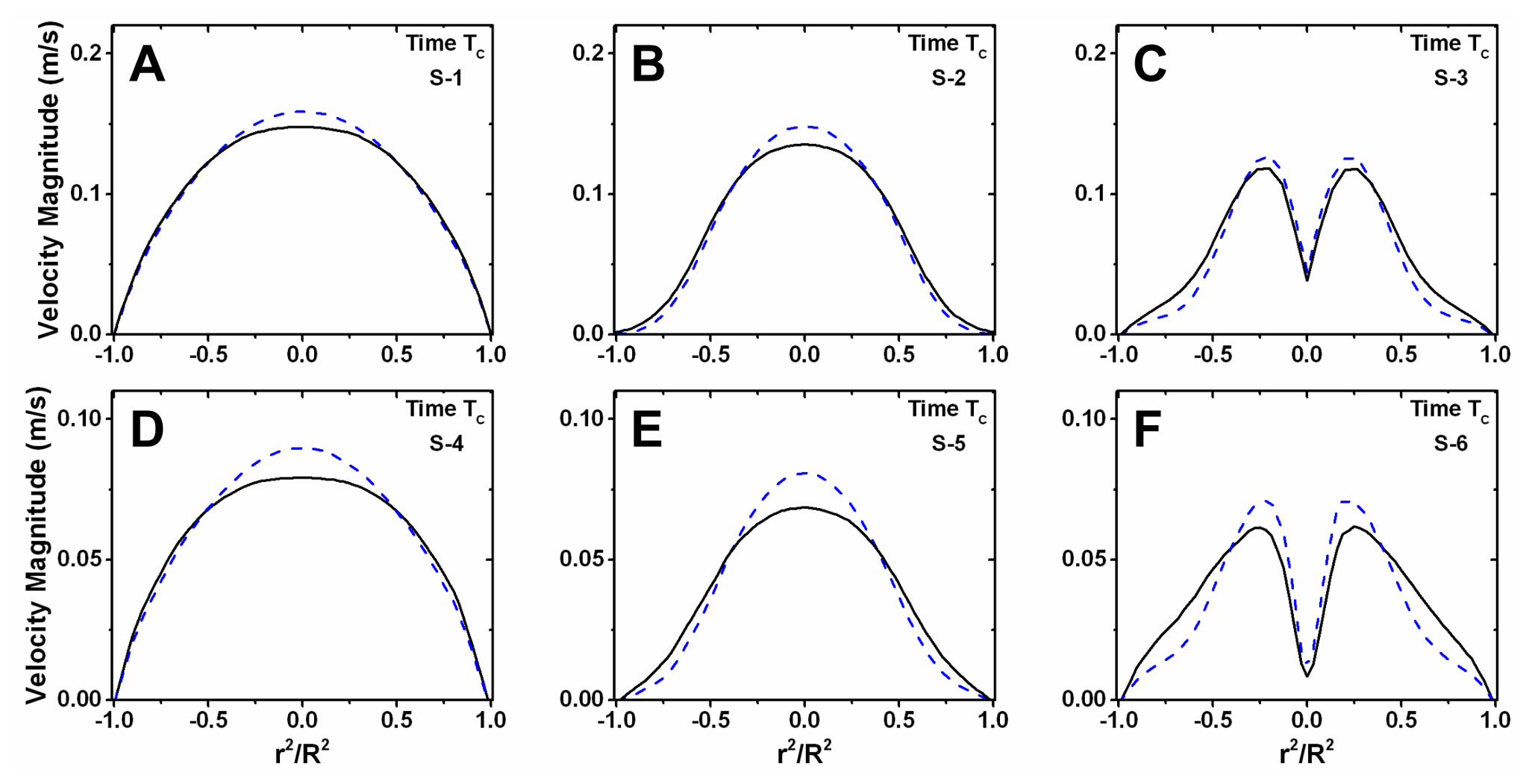

Supplement: S3 Fig — (A) Velocity profiles plotted over the cross sections shown at TC. Velocity slices across first bifurcation were extracted at (B) the bifurcation start, (C) bifurcation midpoint, and (D) the bifurcation apex. Velocity slices were extracted at respective locations (E-G) across the second bifurcation. X-axis is shown as squared distance from the vessel center (r) over the squared vessel radius (R). For slices within the bifurcations (C, D, F, G) the vessel is not cylindrical, so R is chosen as half the wall to wall length. The solid black line is the SDF velocity profile and the dashed blue line is the Newtonian velocity profile. (TIF) [file pone.0124575.s003.tif]
