# Supplementary material for: Hemodynamic Analysis in an Idealized Artery Tree: Differences in Wall Shear Stress between Newtonian and Non-Newtonian Blood Models
Source: PLoS One. 2015 Apr 21;10(4):e0124575. doi: 10.1371/journal.pone.0124575 (PMC4405589; doi:10.1371/journal.pone.0124575)
Supplement: S1 File — Lid-driven flow was applied to 2-dimensional and 3-dimensional cavities, and velocity, pressure, and WSS profiles were compared between the Newtonian and SDF fluid models. Fig A, Numerical Method Verification with the 2-Dimensional Lid-Driven Cavity Flow Problem. Verification of our numerical method using simulations provided by Ghia in the 2-dimensional lid-driven cavity flow problem [44]. (A) Flow profile throughout the cavity from the Newtonian model at Re = 10,000. Vx was extracted along the white line in the geometric center of the cavity at (A) Re = 1,000; (B) Re = 5,000; and (C) Re = 10,000. The bottom of the cavity is defined as y = 0 and the top as y = 1. Fig B, Effect of Cavity Size and Re on Newtonian Velocity Profiles. Effect of the cavity size on the Newtonian velocity profiles across the geometric center of the 2-dimensional cavity at (A) Re = 500, (B) Re = 5,000, and (C) Re = 10,000. The full cavity size is 1m x 1m, the 10% size is 0.1 m x 0.1 m, and the 1% size is 0.01 m x 0.01 m. The y-coordinate of the smaller cavities are scaled up for visualization purposes. Fig C, Effect of Cavity Size and Re on SDF Velocity Profiles. Effect of the cavity size on the SDF velocity profiles across the geometric center of the 2-dimensional cavity at (A) Re = 500, (B) Re = 5,000, and (C) Re = 10,000. The full cavity size is 1m x 1m, the 10% size is 0.1 m x 0.1 m, and the 1% size is 0.01 m x 0.01 m. The y-coordinate of the smaller cavities are scaled up for visualization purposes. Fig D, Pressure and Velocity Profiles in the 3-Dimensional Cavity. (A) The 3-dimensional cavity (1m x 1m x 1m) with inflow applied across the top of the cavity in the positive x-direction. (B) Pressure and (C) Vx profiles were extracted along the red line parallel to the y-axis shown. The bottom of the cavity is defined as y = 0 and the top of the cavity as y = 1. Fig E, WSS and Wall Pressure along the 3-Dimensional Cavity. (A) WSS across the 3-dimensional cavity for the SDF (left) and Newt [file pone.0124575.s004.docx]

*2-dimensional Lid-Driven Cavity Flow Numerical Simulations*

Our numerical method gives excellent agreement to the benchmark lid-driven cavity flow problem published by Ghia and colleagues [1]. The cavity has length and width dimensions of 1 m x 1 m (denoted full sized), and a tangential velocity was introduced across the top of the cavity in the positive x-direction (Fig. A). The Reynolds numbers (*Re*) is expressed as:

$\mathrm{Re}=\frac{\rho VL}{\mu}$ (1)

where *ρ* is the fluid density, *V* is the driving velocity, $\mu$ is the fluid viscosity, and *L* is the cavity height. We extracted the x-component of the velocity (V_x_) along the y-axis in the geometric center of e cavity across *Re* of 1,000; 5,000; and 10,000.


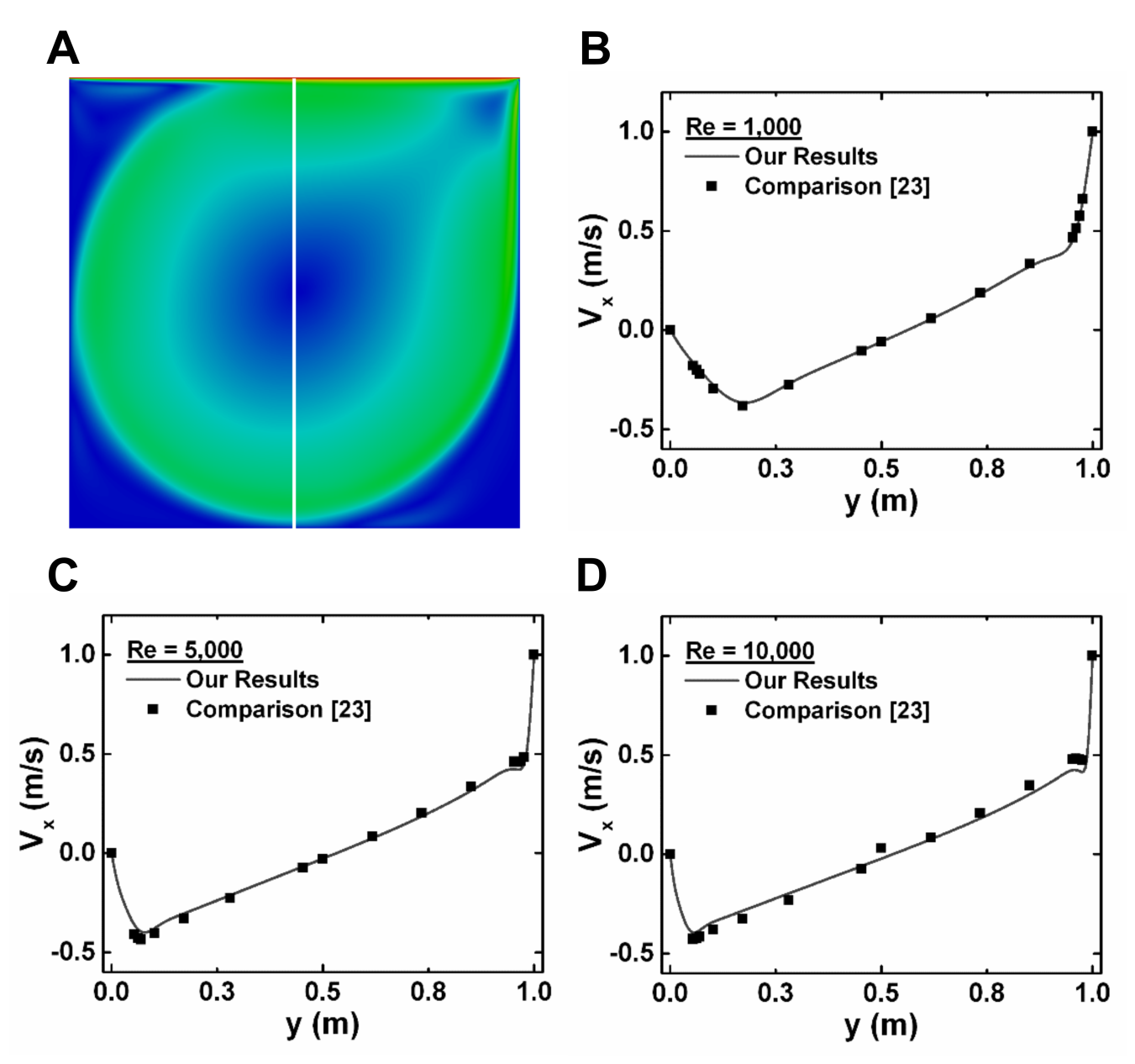


**Fig. A.** **Numerical Method Verification with the 2-Dimensional Lid-Driven Cavity Flow Problem.** Verification of our numerical method using simulations provided by Ghia in the 2-dimensional lid-driven cavity flow problem [44]. (A) Flow profile throughout the cavity from the Newtonian model at *Re* = 10,000. V_x_ was extracted along the white line in the geometric center of the cavity at (A) *Re* = 1,000; (B) *Re =* 5,000; and (C) *Re* = 10,000. The bottom of the cavity is defined as y = 0 and the top as y = 1.

Given the same *Re*, the Newtonian model gives similar velocity profiles through the 2-dimensional cavity at different height and width scales, whereas the SDF gives notably different velocity profiles. The cavity was made to three sizes: (i) the full size cavity (1 m x 1 m), (ii) with height and width 10% of the full size (0.1 m x 0.1 m), and (iii) with height and width 1% of the full size (0.01 m x 0.01 m). Simulations were performed using three *Re* for each cavity size (*Re =* 500; 5,000; and 10,000), chosen as a representative range of *Re* seen in arterial blood flow [2].

**Remark 1:** The *Re* is not constant throughout the cavity with the SDF model, due to the SDF viscosity being inconsistent across the cavity. Thus, for the sake of comparison with the Newtonian model, the same inflow velocity giving a desired *Re* with the Newtonian model was applied to SDF model simulations.

The Newtonian velocity profiles are identical for each cavity size at every *Re* (Fig. B), whereas the SDF velocity profiles largely differ amongst the cavity sizes at low *Re*, but become more similar as the *Re* is increased (Fig. C). The spatial location of lowest magnitude V_x_ in the SDF velocity profiles is noticeably shifted compared to the Newtonian model (Table A). Additionally, the lowest V_x_ magnitude increases at different rates with increasing *Re* for the two models. The Newtonian model gives a 23.7-fold increase in the lowest V_x_ magnitude from *Re* = 500 to *Re* = 10,000 whereas the SDF models gives a 28.9-fold increase in the full size cavity, a 39.1-fold increase in the 10% cavity, and a 30.7-fold increase in the 1% cavity (Table A).


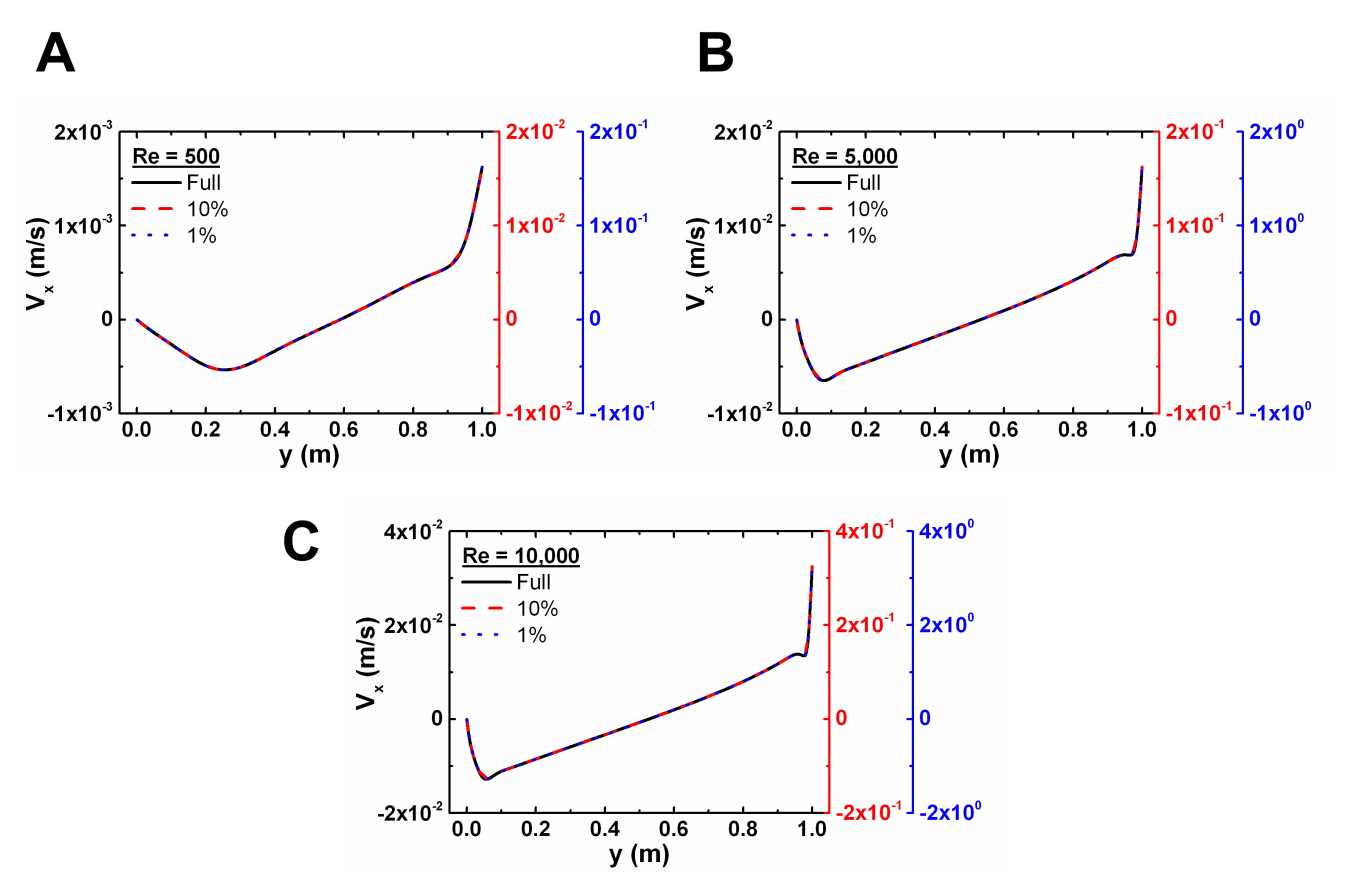


**Fig. B. Effect of Cavity Size and *Re* on Newtonian Velocity Profiles.** Effect of the cavity size on the Newtonian velocity profiles across the geometric center of the 2-dimensional cavity at (A) *Re* = 500, (B) *Re* = 5,000, and (C) *Re* = 10,000. The full cavity size is 1m x 1m, the 10% size is 0.1 m x 0.1 m, and the 1% size is 0.01 m x 0.01 m. The y-coordinate of the smaller cavities are scaled up for visualization purposes.


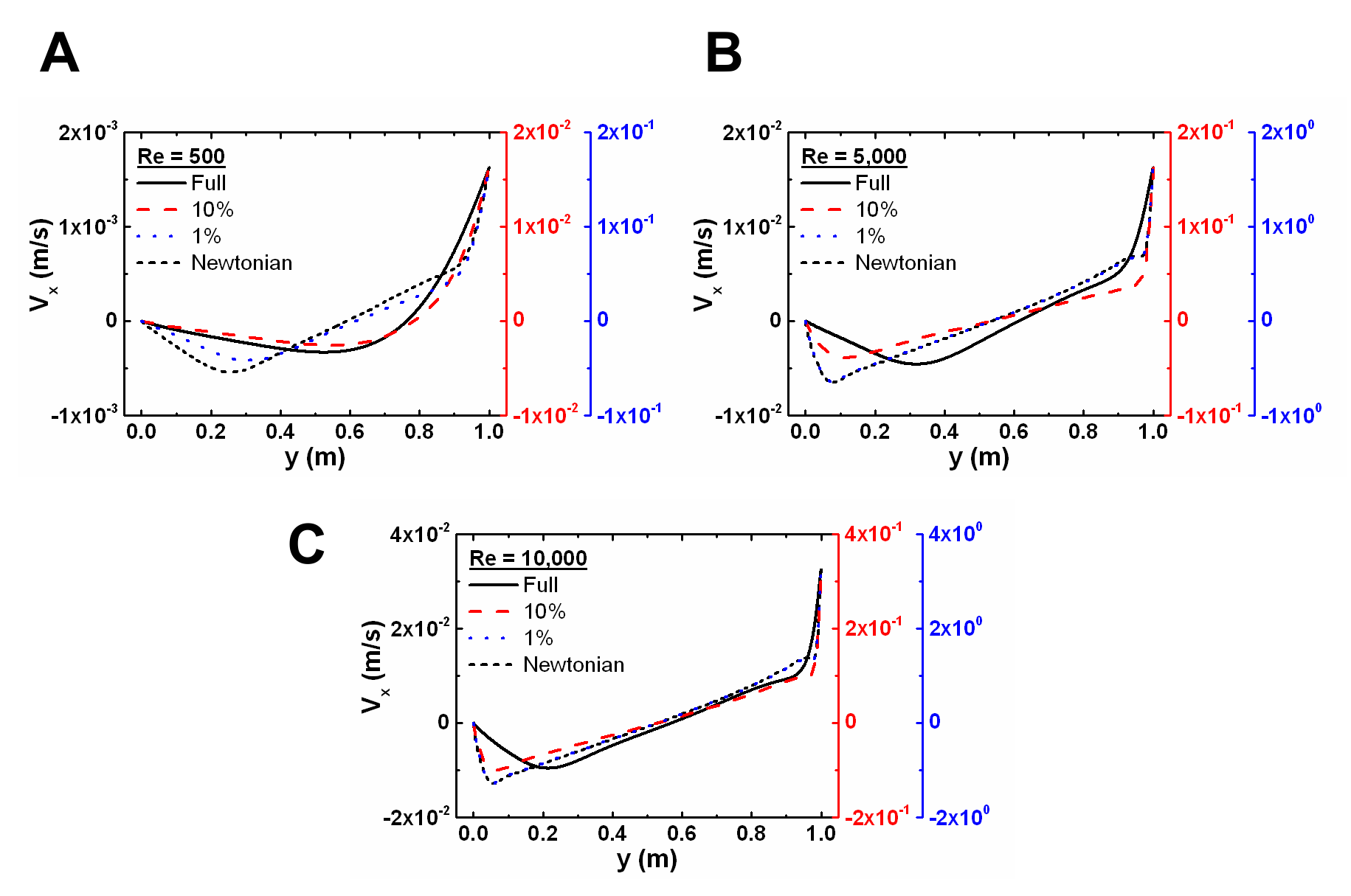


**Fig. C. Effect of Cavity Size and *Re* on SDF Velocity Profiles.** Effect of the cavity size on the SDF velocity profiles across the geometric center of the 2-dimensional cavity at (A) *Re* = 500, (B) *Re* = 5,000, and (C) *Re* = 10,000. The full cavity size is 1m x 1m, the 10% size is 0.1 m x 0.1 m, and the 1% size is 0.01 m x 0.01 m. The y-coordinate of the smaller cavities are scaled up for visualization purposes.

**Table A. The lowest V_x_ magnitude for the 2-dimensional cavity at different sizes.**

| Cavity Type | V_x_ (mm/s) at  *Re* = 500 | V_x_ (mm/s) at  *Re* = 5,000 | V_x_ (mm/s) at  *Re* = 10,000 |
| --- | --- | --- | --- |
| Newtonian | -0.54 | -6.50 | -12.8 |
| SDF (Full) | -0.33 | -4.56 | -9.55 |
| SDF (10%) | -2.55 | -38.5 | -99.82 |
| SDF (1%) | -41.52 | -640.1 | -1273.10 |

The lowest V_x_ magnitude in mm/s along the geometric center of the 2-dimensional cavity for the Newtonian and SDF models. The bottom of the cavity is defined as y = 0 m and the top as y = 1 m. Only one cavity size is shown for the Newtonian model.

We note distinct kinks, points of rapid slope change, in the velocity profiles (Fig. B, C). The spatial location of these kinks given by the SDF model is noticeably different for the different cavity sizes (Table B). However, the kinks given by the SDF model converge to those of the Newtonian model as the cavity size is decreased or the *Re* is increased (Table B).

**Table B. Spatial locations of kinks in V_x_ profiles in the 2-dimensional cavity at different sizes.**

| Cavity Type | Kink location (m) at  *Re* = 500 | | Kink location (m) at *Re* = 5,000 | | Kink location (m) at *Re* = 10,00 | |
| --- | --- | --- | --- | --- | --- | --- |
|  | First | Second | First | Second | First | Second |
| Newtonian | 0.26 | 0.89 | 0.08 | 0.95 | 0.06 | 0.97 |
| SDF (Full) | 0.52 | - | 0.32 | 0.86 | 0.21 | 0.88 |
| SDF (10%) | 0.54 | - | 0.11 | 0.94 | 0.06 | 0.95 |
| SDF (1%) | 0.31 | 0.89 | 0.08 | 0.95 | 0.06 | 0.97 |

The location of kinks in the V_x_ profile from the bottom of the cavity along the geometric center for the Newtonian and SDF models. Kinks are seperated by a semi-colon for that particular *Re*; if only one kink is present the second is labeled as “-“. Only one cavity size is shown for the Newtonian model.

*3-dimensional Lid-Driven Cavity Flow Numerical Simulations*

The benchmark 2-dimensional lid-driven cavity problem was extended to three dimensions. Simulations were performed to investigate the third dimensions impact on velocity, pressure, and WSS given by the Newtonian and SDF models. The driving velocity was again applied across the top of the cavity in the positive x-direction.

The SDF model gives a larger pressure drop across the 3-dimensional cavity than the Newtonian model. Pressure and V_x_ were extracted along a line parallel to the y-axis in the geometric middle of the cavity (Fig. D). The Newtonian model displays a more fully developed circular flow profile than the SDF model, and the lowest V_x_ magnitude is 1.15-fold larger in the Newtonian model. Pressure profiles reveal that the SDF model has a 2.87-fold larger pressure drop across the cavity than the Newtonian model (Table C).


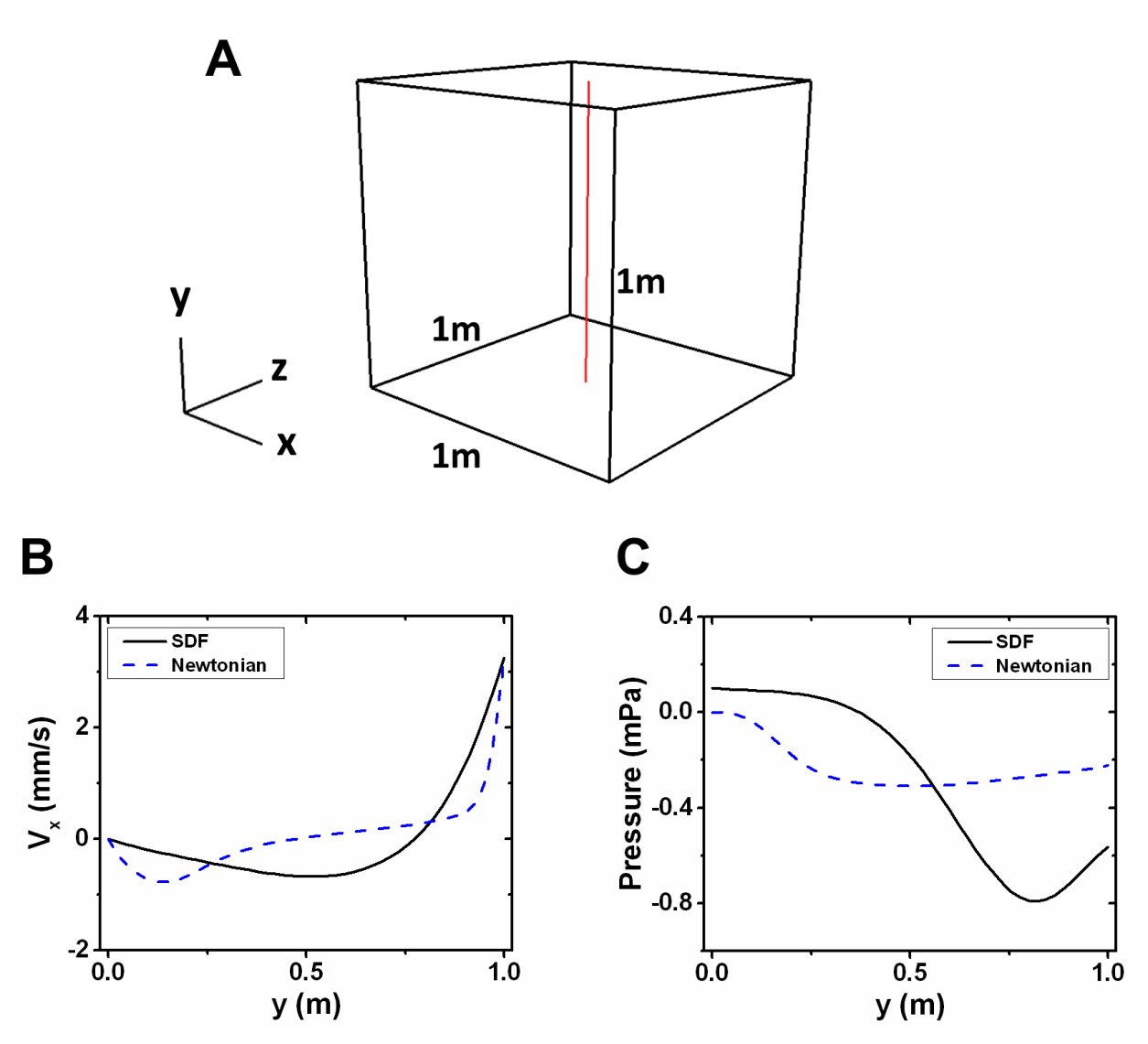


**Fig. D. Pressure and Velocity Profiles in the 3-Dimensional Cavity.** (A) The 3-dimensional cavity (1m x 1m x 1m) with inflow applied across the top of the cavity in the positive x-direction. (B) Pressure and (C) V_x_ profiles were extracted along the red line parallel to the y-axis shown. The bottom of the cavity is defined as y = 0 and the top of the cavity as y = 1.

**Table C. Velocity and pressure differences between the two models in the 3-dimensional cavity.**

|  | Lowest V_x_ (mm/s) | Lowest V_x_ Location (m) | Lowest Pressure (mPa) | Lowest Pressure Location (m) | Highest  Pressure (mPa) |
| --- | --- | --- | --- | --- | --- |
| Newtonian | -0.78 | 0.14 | -0.31 | 0.50 | 0.00 |
| SDF | -0.68 | 0.51 | -0.79 | 0.82 | 0.10 |

The lowest V_x_ magnitude and spatial location, lowest pressure magnitude and spatial location, and highest press magnitude for the Newtonian and SDF models across the geometric center of the 3-dimensional cavity at *Re* = 1,000. Spatial location are given in distance from the bottom of the cavity along the geometric center of the cavity. Highest pressure spatial location is always located at the top of the cavity, and is thus omitted.

The SDF model exhibits an equal or higher WSS magnitude at every spatial location across the 3-dimensional cavity compared to the Newtonian model (Fig. E). Along the center of the right cavity wall, the SDF model gives 7.3-fold larger WSS and 4.1-fold larger pressure than the Newtonian model at *Re* = 1,000. Exponential growth curve fits show that WSS and pressure increase more rapidly with the SDF model than the Newtonian model.

**
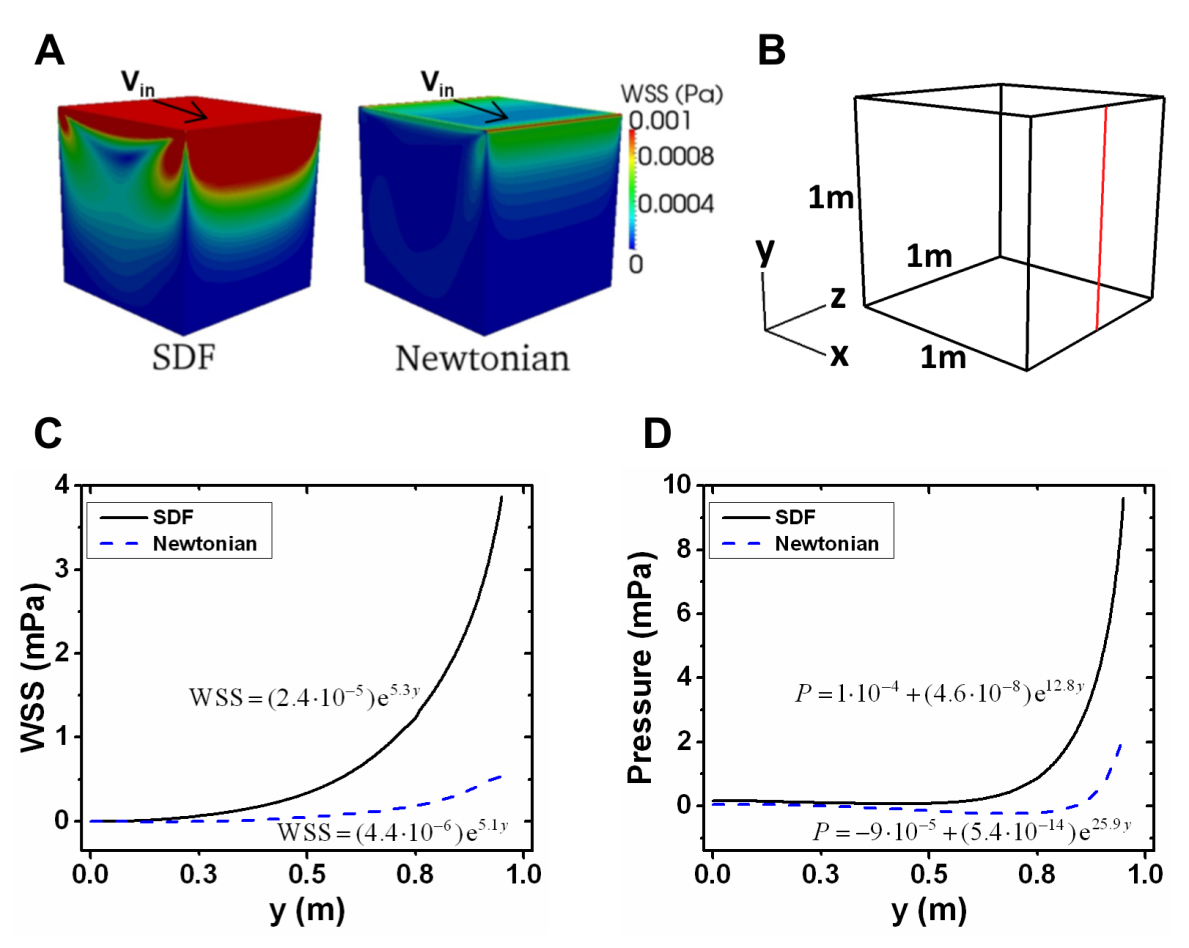
**

**Fig. E.** **WSS and Wall Pressure along the 3-Dimensional Cavity.** (A) WSS across the 3-dimensional cavity for the SDF (left) and Newtonian (right) models. (B) The 3-dimensional cavity where (C) WSS and (D) wall pressure were extracted along the red line parallel to the y-axis and along the right wall. The bottom of the cavity is defined as y = 0 and the top of the cavity is y = 1.

**References**

1.     Ghia U, Ghia KN, Shin CT. High-Re solutions for incompressible flow using the Navier-Stokes equations and a multigrid method. J Comput Phys. 1982;48: 387-411.

2.     Ku D. Blood flow in arteries. Annu Rev Fluid Mech. 1997;29: 399-434.
