# Supplementary material for: Hemodynamic Analysis in an Idealized Artery Tree: Differences in Wall Shear Stress between Newtonian and Non-Newtonian Blood Models
Source: PLoS One. 2015 Apr 21;10(4):e0124575. doi: 10.1371/journal.pone.0124575 (PMC4405589; doi:10.1371/journal.pone.0124575)
Supplement: S1 Table — The element type used was either the 8-node hexahedral (HEX8) or the 10-node tetrahedral (TET10) element. Note that the 2-dimensional cavity was created as a 3-dimensional geometry using HEX8 elements with the cavity thickness (z-dimension) being 20% of the height and width (0.05 m for the full size cavity). However, the z-directional velocity was held constant at zero to produce 2-dimensional physics from a 3-dimensional geometry. We conducted the 2-dimensional cavity simulations in this way because our numerical method provides greater stability and convergence rates for 3-dimensional element types. All geometries except for the idealized femoral artery tree were simulated at steady state, and thus, given the chosen initial velocity, the number of time steps taken were used to reach the desired Re. The idealized femoral artery tree was simulated transiently, and 200 time steps were chosen to simulate the time course of 2 seconds. The Re indicated for the idealized femoral artery tree is the mean Re through the parent artery over the inflow velocity waveform. (DOCX) [file pone.0124575.s005.docx]

**S1 Table. Numerical parameters for simulations of each geometry used in this study.**

| Geometry | Element type | Number of elements | Number of nodes | Time step size (s) | Number of time steps | Initial velocity (m/s) | *Re* simulated |
| --- | --- | --- | --- | --- | --- | --- | --- |
| 2-dimensional cavity | HEX8 | 25,600 | 51,842 | 50 | 20 | 3.25 x 10^-5^ | 10,000 |
| 3-dimensional cavity | HEX8 | 262,144 | 274,625 | 1 | 100 | 3.25 x 10^-5^ | 1,000 |
| Single bifurcating artery | TET10 | 21,802 | 33,883 | 5 | 195 | 1.68 x 10^-3^ | 505 |
| Idealized femoral artery tree | TET10 | 173,419 | 264,564 | 0.01 | 200 | 1.60 x 10^-1^ | 550 |

The element type used was either the 8-node hexahedral (HEX8) or the 10-node tetrahedral (TET10) element. Note that the 2-dimensional cavity was created as a 3-dimensional geometry using HEX8 elements with the cavity thickness (z-dimension) being 20% of the height and width (0.05 m for the full size cavity). However, the z-directional velocity was held constant at zero to produce 2-dimensional physics from a 3-dimensional geometry. We conducted the 2-dimensional cavity simulations in this way because our numerical method provides greater stability and convergence rates for 3-dimensional element types. All geometries except for the idealized femoral artery tree were simulated at steady state, and thus, given the chosen initial velocity, the number of time steps taken were used to reach the desired *Re*. The idealized femoral artery tree was simulated transiently, and 200 time steps were chosen to simulate the time course of 2 seconds. The *Re* indicated for the idealized femoral artery tree is the mean *Re* through the parent artery over the inflow velocity waveform.
